# The CMSSM and NUHM1 after LHC Run 1

**DOI:** 10.1140/epjc/s10052-014-2922-3

**Published:** 2014-06-13

**Authors:** O. Buchmueller, R. Cavanaugh, A. De Roeck, M. J. Dolan, J. R. Ellis, H. Flächer, S. Heinemeyer, G. Isidori, J. Marrouche, D. Martínez Santos, K. A. Olive, S. Rogerson, F. J. Ronga, K. J. de Vries, G. Weiglein

**Affiliations:** 1 High Energy Physics Group, Blackett Laboratory, Imperial College, Prince Consort Road, London, SW7 2AZ UK; 2 Fermi National Accelerator Laboratory, P.O. Box 500, Batavia, IL 60510 USA; 3 Physics Department, University of Illinois at Chicago, Chicago, IL 60607-7059 USA; 4 Physics Department, CERN, 1211 Geneve 23, Switzerland; 5 Antwerp University, 2610 Wilrijk, Belgium; 6 Theory Group, SLAC National Accelerator Laboratory, 2575 Sand Hill Road, Menlo Park, CA 94025-7090 USA; 7 Theoretical Particle Physics and Cosmology Group, Department of Physics, King’s College London, London, WC2R 2LS UK; 8 H.H. Wills Physics Laboratory, University of Bristol, Tyndall Avenue, Bristol, BS8 1TL UK; 9 Instituto de Física de Cantabria (CSIC-UC), 39005 Santander, Spain; 10 INFN, Laboratori Nazionali di Frascati, Via E. Fermi 40, 00044 Frascati, Italy; 11 NIKHEF and VU University Amsterdam, Science Park 105, 1098 XG Amsterdam, The Netherlands; 12 William I. Fine Theoretical Physics Institute, School of Physics and Astronomy, University of Minnesota, Minneapolis, MN 55455 USA; 13 Institute for Particle Physics, ETH Zürich, 8093 Zurich, Switzerland; 14 DESY, Notkestrasse 85, 22607 Hamburg, Germany

## Abstract

We analyze the impact of data from the full Run 1 of the LHC at 7 and 8 TeV on the CMSSM with $\mu > 0$ and $<0$ and the NUHM1 with $\mu > 0$, incorporating the constraints imposed by other experiments such as precision electroweak measurements, flavour measurements, the cosmological density of cold dark matter and the direct search for the scattering of dark matter particles in the LUX experiment. We use the following results from the LHC experiments: ATLAS searches for events with ${E\!\!/}_{T}$ accompanied by jets with the full 7 and 8 TeV data, the ATLAS and CMS measurements of the mass of the Higgs boson, the CMS searches for heavy neutral Higgs bosons and a combination of the LHCb and CMS measurements of $\mathrm{BR}(B_s \rightarrow \mu ^+\mu ^-)$ and $\mathrm{BR}(B_d \rightarrow \mu ^+\mu ^-)$. Our results are based on samplings of the parameter spaces of the CMSSM for both $\mu >0$ and $\mu <0$ and of the NUHM1 for $\mu > 0$ with 6.8$\times 10^6$, 6.2$\times 10^6$ and 1.6$\times 10^7$ points, respectively, obtained using the MultiNest tool. The impact of the Higgs-mass constraint is assessed using FeynHiggs 2.10.0, which provides an improved prediction for the masses of the MSSM Higgs bosons in the region of heavy squark masses. It yields in general larger values of $M_h$ than previous versions of FeynHiggs, reducing the pressure on the CMSSM and NUHM1. We find that the global $\chi ^2$ functions for the supersymmetric models vary slowly over most of the parameter spaces allowed by the Higgs-mass and the ${E\!\!/}_{T}$ searches, with best-fit values that are comparable to the $\chi ^2/\mathrm{dof}$ for the best Standard Model fit. We provide 95 % CL lower limits on the masses of various sparticles and assess the prospects for observing them during Run 2 of the LHC.

## Introduction

In addition to establishing the mechanism for electroweak symmetry breaking, one of the primary objectives of experiments at the LHC has been to search for possible physics beyond the Standard Model (SM), such as new particles that might alleviate the naturalness problem and/or be associated with cosmological dark matter. In contrast with the triumphant discovery at the LHC of a particle that resembles the Higgs boson of the SM [1, 2], and the observation of $\mathrm{BR}(B_s \rightarrow \mu ^+\mu ^-)$ decay at a rate close to the SM prediction [3, 4], the first run of the LHC has not revealed any convincing evidence for physics beyond the SM. In particular, the LHC searches for jets + ${E\!\!/}_{T}$ events [5, 6] and for heavy Higgs bosons $H^\pm /H/A$ [7] have drawn blanks so far. In parallel, neither direct nor indirect searches for astrophysical dark matter have found any convincing signals [8, 9], posing questions regarding the implications of those results for supersymmetric models.

We have published previously several analyses of constrained versions of the minimal supersymmetric extension of the Standard Model (MSSM) with universal soft supersymmetry (SUSY)-breaking parameters $m_0$ for scalars and $m_{1/2}$ for fermions as well as a trilinear coupling $A_0$ at an input grand unification scale and $\tan \beta$, the ratio of the two vacuum expectation values at the electroweak scale (the CMSSM [10–20]). We have also analyzed its generalisation to include common but non-universal soft supersymmetry-breaking Higgs masses $m_H$ (the NUHM1 [20–23]). We have analyzed these models both before the start-up of the LHC and in the contexts of successive releases of LHC data [24–33].

Prior to the LHC start-up, the discrepancy between the experimental measurement of $(g-2)_\mu$ [34, 35] and theoretical calculations (see [36, 37] and references therein), favoured relatively light sparticle masses, but these have not appeared in ATLAS and CMS ${E\!\!/}_{T}$ searches, disfavouring small values of the CMSSM or NUHM1 SUSY-breaking mass parameters [5]. On the other hand, the discovery of a SM-like Higgs boson by ATLAS and CMS [1, 2] with a mass consistent with the predictions of SUSY models has provided an important indirect constraint on SUSY model parameters such as $m_0, m_{1/2}$, $A_0$ and $\tan \beta$. A significant rôle is also played by the observation by CMS and LHCb of $\mathrm{BR}(B_s \rightarrow \mu ^+\mu ^-)$ decay [3, 4], which imposes a complementary constraint on the CMSSM and NUHM1 parameter spaces. Our most recent analyses [33] of these models were based on the 7-TeV ATLAS 5/fb jets + ${E\!\!/}_{T}$ data set [38, 39]. In this paper we update our analyses to include the 8-TeV ATLAS 20/fb jets + ${E\!\!/}_{T}$ data set [5], providing a complete study of the implications of the LHC Run 1 for the CMSSM and NUHM1 scenarios. On the basis of this study, we also discuss the prospects for discovering sparticles in the LHC Run 2.

As described below, these constraints are analyzed in a frequentist approach using an overhauled version of the MasterCode [40] framework to calculate the global $\chi ^2$ function. For other recent post-LHC analyses of the CMSSM and NUHM, see [41–66]. In this paper we sample the CMSSM and NUHM1 parameter spaces using the MultiNest tool [67, 68], which is more efficient than the Markov Chain Monte Carlo technique we used previously. We implement the ATLAS 20/fb jets + ${E\!\!/}_{T}$ constraint using scaling laws to extrapolate the sensitivity to regions of the parameter space where documentation is not available [33]. In our implementation of the $M_h$ constraint we use a new version of FeynHiggs, 2.10.0 [69], which incorporates a resummation of leading and subleading logarithmic corrections from the top/stop sector to provide improved results for larger stop masses. Since in the SUSY models we study $\mathrm{BR}(B_s \rightarrow \mu ^+\mu ^-)$ and $\mathrm{BR}(B_d \rightarrow \mu ^+\mu ^-)$ are expected to have the same ratio as in the SM, we combine these constraints by assuming this SM ratio and incorporating the experimental correlations between $\mathrm{BR}(B_s \rightarrow \mu ^+\mu ^-)$ and $\mathrm{BR}(B_d \rightarrow \mu ^+\mu ^-)$ reported by the LHCb and CMS Collaborations [3, 4]. Recent cosmological observations, including those by the Planck satellite [70], have refined the estimate of the cold dark matter density, but this does not have a relevant impact on our study. Concerning direct searches for dark matter, the only constraint we apply is that from LUX [9] on spin-independent dark matter scattering, which we incorporate taking due account of the uncertainties in the hadronic scattering matrix element, as discussed later.

We find that the global $\chi ^2$ function varies relatively little across most of the regions of the $(m_0, m_{1/2})$ planes that are allowed by the ${E\!\!/}_{T}$, Higgs and dark matter density constraints on the CMSSM and NUHM1 parameter spaces, with a global minimum at large $m_0$ and $m_{1/2}$, which is similar to the $\chi ^2/\mathrm{dof}$ for the SM. Within the CMSSM, there are four principal mechanisms for bringing the SUSY relic density $\Omega _\chi h^2$ into the range favoured by Planck and other measurements [70], namely coannihilation with the lighter stau $\tilde{\tau }_1$ and other sleptons, coannihilation with the lighter stop ${\widetilde{t}_1}$, rapid annihilation through the heavy Higgs bosons $H, A$ in the direct channel and annihilation in the focus-point region where the lightest neutralino $\tilde{\chi }^0_{1}$ has an enhanced Higgsino component. In the following, we comment on the respective rôles of these mechanisms.^1^ Within the range of the CMSSM parameter space examined in this paper, the $\Omega _\chi h^2$ constraint sets an upper bound on $m_{1/2}$ but not on $m_0$. In the case of the NUHM1, more annihilation mechanisms may come into play, and we find no upper bound on either $m_0$ or $m_{1/2}$.

One of our key findings is that the LHC measurement of $M_h$ is not in tension with other constraints on the CMSSM and NUHM1 parameter spaces except for $(g-2)_\mu$. The $M_h$ constraint does not impact them as strongly as had previously been thought [62, 63], since the improved prediction incorporated in FeynHiggs 2.10.0 [69] yields in general a higher value of $M_h$ than previous versions of FeynHiggs (as well as SoftSUSY) for the same values of the model parameters [64], as will be discussed in detail in Sect. 2.4. The best-fit point in the CMSSM with $\mu > 0$ ($< 0$) has $\tan \beta \sim 51 (36)$, and $\tan \beta \sim 39$ is preferred in the NUHM1 with $\mu > 0$. All these points have relatively large values of $m_0$ and $m_{1/2}$, but the likelihood functions of these models are quite flat, and each of the models also has a local minimum of the likelihood function at low mass, with smaller $\tan \beta$ and small $\Delta \chi ^2 \le 1$ relative to the global minimum. We present 95 % CL lower limits on $m_{\tilde{g}}$ (the gluino mass), $m_{\tilde{q}_R}$ (the average over the right-handed squark masses of the first two generations), $m_{\tilde{t}_1}$ (the light scalar top mass) and $m_{\tilde{\tau }_1}$ (the light scalar tau mass) in each of these models. In each case, we find that the lighter stop ${\tilde{t}_1}$ may be significantly lighter than the other strongly interacting sparticles.

The structure of this paper is as follows. In Sect. 2 we discuss the updated MasterCode framework and the more important changes in our implementations of the experimental constraints. There are no significant changes in the ways we treat the constraints not discussed explicitly. In Sect. 3 we describe the results of our fits within the CMSSM and NUHM1. Finally, in Sect. 4 we summarise our conclusions and discuss the prospects for future studies of these and other SUSY models, in particular during the LHC Run 2.

## Implementations of the principal experimental constraints

### The Mastercode framework

As described in our previous papers [24–33], the MasterCode [40] is a framework that incorporates a code for the electroweak observables based on [71, 72]^2^ as well as the SoftSUSY 3.3.9 [74], FeynHiggs 2.10.0 [69, 75–79], SuFla [80, 81], SuperIso 3.3 [82–84], MicrOMEGAs 3.2 [85–87] and SSARD [88] codes, which are interfaced using the SUSY Les Houches Accord [89, 90]. The MasterCode is used to construct a global likelihood function that includes contributions from electroweak precision observables, flavour measurements, the cosmological dark matter density and direct searches for dark matter, as well as the LHC Higgs-mass measurement and ${E\!\!/}_{T}$ searches.

### Implementation of MultiNest

There has been a major overhaul of the MasterCode since [33], with the aim of simplifying its use and facilitating its application to different SUSY models. The most important change in its implementation has been to use the MultiNest algorithm [67, 68] to sample parameter spaces, instead of the Markov Chain Monte Carlo (MCMC) approach used previously. We find that MultiNest is significantly more efficient for our purposes, and we have extensively checked that results obtained using the new version of the MasterCode agree with those obtained from the previous version when the same input constraints are used.

Although MultiNest, like other sampling techniques such as MCMC, is geared towards Bayesian interpretation approaches, it can be used to sample well multi-dimensional parameter spaces, and thereby estimate efficiently and robustly frequentist confidence intervals. The main requirements for our purposes are that no nodes of high likelihood are missed, and that the regions with low $\chi ^2$ are well sampled. For the scans used in this paper we use the ranges $0 < m_0 < 7000 \,\, \mathrm {GeV}$, $0 < m_{1/2} < 4000 \,\, \mathrm {GeV}$, $2 < \tan \beta < 68$ and $- 5000 \,\, \mathrm {GeV}< A_0 < 5000 \,\, \mathrm {GeV}$ 3 in the CMSSM, for both signs of $\mu$, thereby extending significantly the $m_0$ range compared to [33]. In the case of the NUHM1, we use the same ranges for $m_{1/2}, \tan \beta$ and $A_0$, sample $0<m_0<4000$ and study the range $- 5\times 10^7 \,\, \mathrm {GeV}^2 < m_{H}^2 < 5\times 10^7 \,\, \mathrm {GeV}^2$, restricting our attention to $\mu > 0$. The total numbers of points sampled in the CMSSM with $\mu >0$ and $\mu <0$ and the NUHM1 are 6.8$\times 10^6$, 5.3$\times 10^6$ and 1.6$\times 10^7$, respectively. In all cases, the best-fit points were checked by running Minuit [91] on the parameter space, and the differences in total $\chi ^2$ between MultiNest and Minuit were $\ll 1$ %.

In this analysis we make several changes in our implementations of the constraints, of which the most important are described in the following subsections.

### The ATLAS 20/fb jets + ${E\!\!/}_{T}$ constraint

The ATLAS Collaboration has made public preliminary updates of their SUSY searches using the entire available 8 TeV data set, including the results of many different searches targeting different ${E\!\!/}_{T}$ final states and topologies. Here we follow the same prescription as in [33], restricting ourselves to using the 0-lepton + 2 to 6 jets + ${E\!\!/}_{T}$ search [5]. This is done in order to ensure that the limits presented by ATLAS in the CMSSM ${m_0, m_{1/2}}$ plane for $\tan \beta = 30$ and $A_0 = 2 m_0$ can be extrapolated to other values of $\tan \beta$ and $A_0$ in the ranges used in our scan. As in [33], we have performed a dedicated validation to check that the 0-lepton ${E\!\!/}_{T}$ limit reported in [5] is quite independent of $\tan \beta$ and $A_0$. As was to be to be anticipated given the similarity of the search methodologies between the ATLAS 0-lepton analyses at 7 and 8 TeV, we find very similar results to [33]. Therefore, we assume that the 95 % CL exclusion contour in the $(m_0, m_{1/2})$ plane presented in [5] may be used irrespective of $\tan \beta$ and $A_0$, and we apply a penalty term to points in our scan according to their distance from the stated 95 % CL limit, using the same scaling function as in [33].

We do not use other LHC SUSY searches (such as those relying on lepton and/or $b$-jet signatures) in our analysis, for two reasons. One is that in the CMSSM and associated models the jet + ${E\!\!/}_{T}$ searches give the strongest constraints in the most relevant region of parameter space, as we discuss in more detail below. The other is that these are more sensitive to the model parameters, whereas the 0-lepton + jets + ${E\!\!/}_{T}$ search sensitivity is known [33] to be independent of the details of the models we study.

### The Higgs mass constraint

In view of the relatively large value of the Higgs mass [92, 93], $M_h= 125.7 \pm 0.4 \,\, \mathrm {GeV}$ (where the quoted uncertainty is purely experimental) and the stronger lower limits on sparticle masses from direct LHC searches [5] within the CMSSM and NUHM1, the calculation of the Higgs boson masses using FeynHiggs has been improved [69] to achieve a higher accuracy for large stop-mass scales. The calculations implemented in FeynHiggs 2.8.7, which we used previously^4^ included the full one-loop contributions and the leading and subleading two-loop corrections. The calculations included in the new version FeynHiggs 2.10.0 used here [69] include a resummation to all orders of the leading and next-to-leading logarithms of the type $\log (m_{\widetilde{t}}/m_t)$ (where $m_{\widetilde{t}}$ denotes the geometric average of the two scalar top masses), based on the relevant two-loop Renormalisation-Group Equations (RGEs) [94]; see [95] and references therein for details. The effects of this new correction start at the three-loop order. It has been ensured that the resummed logarithms, which are obtained in the ${\overline{\mathrm{MS}}}$ scheme, are correctly matched onto the one- and two-loop corrections in the on-shell scheme that were already included previously [69]. The main effect is an upward shift of $M_h$ for stop masses in the multi-TeV range, as well as the possibility of a refined estimate of the theoretical uncertainty that is incorporated in our global fits. This shift in $M_h$ relaxes substantially the constraints from the Higgs mass on the CMSSM and NUHM1 and related models [64].

A numerical analysis in the CMSSM including leading three-loop corrections to $M_h$ using the code H3m [96–98]) was presented in [99]. It was shown that the leading three-loop terms can have a strong impact on the interpretation of the measured Higgs-mass value in the CMSSM. Here, with the new version of FeynHiggs, we go beyond this analysis by including (formally) subleading three-loop corrections as well as a resummation to all orders of the logarithmic contributions to $M_h$; see above.

The new version of FeynHiggs also includes an updated estimate of the theoretical uncertainty, $\Delta M_h|_\mathrm{FH}$, due to missing higher-ordercontributions to $M_h$ [69], which is typically in the range 1.0 to 1.5 GeV in the favoured regions of the parameter spaces we sample. The theoretical uncertainty is to be incorporated in the global $\chi ^2$ function via a contribution of the form

1 $$\begin{aligned} \Delta \chi ^2 (M_h) \; = \; \frac{(M_{h, \mathrm{FH}} - M_{h, \mathrm{exp}})^2}{(\Delta M_h|_\mathrm{FH})^2 + (\Delta M_h|_\mathrm{exp})^2}. \end{aligned}$$

Conservatively, in this paper we assume a fixed value $\Delta M_h|_\mathrm{FH} = 1.5 \,\, \mathrm {GeV}$ in our evaluation of (1), pending a more complete evaluation of $\Delta M_h|_\mathrm{FH}$ in a future version of FeynHiggs.

We do not include in our analysis the current measurements of Higgs production strengths. The measurements in individual channels currently have accuracies measured in the tens of %, whereas the best-fit models we discuss later make predictions that differ from the SM predictions by only a few %, typically considerably less than the current theoretical uncertainties. For this reason, at the present stage the Higgs measurements do not have important impact on the specific models studied models studied in this paper, though they may be relevant now for other models and in the future also for the CMSSM and related models as the precisions of the Higgs measurements improve.

### The $\mathrm{BR}(B_s \rightarrow \mu ^+\mu ^-)$ and $\mathrm{BR}(B_d \rightarrow \mu ^+\mu ^-)$ constraints

To date, the most precise measurements of $\mathrm{BR}(B_s \rightarrow \mu ^+\mu ^-)$ and $\mathrm{BR}(B_d \rightarrow \mu ^+\mu ^-)$ have been provided by the CMS Collaboration [3]:

2 $$\begin{aligned} \begin{aligned} \mathrm{BR}(B_s \rightarrow \mu ^+\mu ^-)_\mathrm{CMS}&=(3.0_{-0.9}^{+1.0})\times 10^{-9} , \\ \mathrm{BR}(B_d \rightarrow \mu ^+\mu ^-)_\mathrm{CMS}&= (3.5_{-1.8}^{+2.1})\times 10^{-10} , \end{aligned} \end{aligned}$$

and the LHCb Collaboration [4]:

3 $$\begin{aligned} \begin{aligned} \mathrm{BR}(B_s \rightarrow \mu ^+\mu ^-)_\mathrm{LHCb}&= (2.9_{-1.0}^{+1.1})\times 10^{-9} , \\ \mathrm{BR}(B_d \rightarrow \mu ^+\mu ^-)_\mathrm{LHCb}&= (3.7_{-2.1}^{+2.4})\times 10^{-10} . \end{aligned} \end{aligned}$$

These numbers correspond to time averaged (TA) branching fractions,^5^ and are in good agreement with the SM TA expectations [103–105] (see also [106, 107]):

4 $$\begin{aligned} \begin{aligned} \mathrm{BR}(B_s \rightarrow \mu ^+\mu ^-)_\mathrm{SM}&=(3.65\pm 0.23)\times 10^{-9} , \\ \mathrm{BR}(B_d \rightarrow \mu ^+\mu ^-)_\mathrm{SM}&= (1.06\pm 0.09)\times 10^{-10} . \end{aligned} \end{aligned}$$

An official combination of the CMS and LHCb results can be found in the conference note [108]:

5 $$\begin{aligned} \begin{aligned} \mathrm{BR}(B_s \rightarrow \mu ^+\mu ^-)_\mathrm{exp}&=(2.9\pm 0.7)\times 10^{-9} , \\ \mathrm{BR}(B_d \rightarrow \mu ^+\mu ^-)_\mathrm{exp}&= (3.6_{-1.4}^{+1.6})\times 10^{-10} . \end{aligned} \end{aligned}$$

In all new physics (NP) models with minimal flavour violation (MFV) [109, 110], including the CMSSM and the NUHM1, $\mathrm{BR}(B_s \rightarrow \mu ^+\mu ^-)$ and $\mathrm{BR}(B_d \rightarrow \mu ^+\mu ^-)$ can deviate from their corresponding SM predictions, but their ratio remains fixed at the SM value [111, 112]:^6^

6 $$\begin{aligned} \left. \frac{\mathrm{BR}(B_s \rightarrow \mu ^+\mu ^-)_\mathrm{NP}}{\mathrm{BR}(B_d \rightarrow \mu ^+\mu ^-)_\mathrm{NP}} \right| _\mathrm{MFV} = 31.41\pm 2.19 . \end{aligned}$$

We exploit this property to combine $\mathrm{BR}(B_s \rightarrow \mu ^+\mu ^-)$ and $\mathrm{BR}(B_d \rightarrow \mu ^+\mu ^-)$ measurements into a single constraint in the CMSSM (NUHM1) parameter space. In particular, for each of the four measurements in (2) and (3) we determine the ratio

7 $$\begin{aligned} R_{\mu \mu } = \frac{\mathrm{BR}(B_{q} \rightarrow \mu ^+\mu ^-)_\mathrm{exp} }{\mathrm{BR}(B_{q} \rightarrow \mu ^+\mu ^-)_\mathrm{SM}} \quad (q = s, d), \end{aligned}$$

that is independent of $q$ in the context of MFV models.

The four constraints are then combined into a single weighted mean (hereafter denoted $R_{\mu \mu }^\mathrm{exp}$), taking into account the correlations between the different measurements. It should also be noted that SuFla computes directly a theoretical prediction for $R_{\mu \mu }$, allowing one to separate the theory uncertainties into three sources: SUSY theory uncertainties (which are negligible), the uncertainty from (6), and those affecting the SM prediction of the branching fractions.

The CMS Collaboration has provided an estimate of $R_{\mu \mu }^\mathrm{CMS} = 1.01_{-0.26}^{+0.31}$ [113] by combining its $\mathrm{BR}(B_s \rightarrow \mu ^+\mu ^-)$ and $\mathrm{BR}(B_d \rightarrow \mu ^+\mu ^-)$ measurements (and using the SM values for the branching ratios in [106, 107]). Here we construct a joint likelihood for the four measurements (2)–(3) using correlation coefficients between $\mathrm{BR}(B_s \rightarrow \mu ^+\mu ^-)$ and $\mathrm{BR}(B_d \rightarrow \mu ^+\mu ^-)$ of $-50~\%~$ in CMS and $+3~\%~$ in LHCb [114]. In order to do that, we use Eqs. (3) and (4) of [108], where the uncertainty coming from the ratio of hadronisation fractions of the b quark (the ratio of probabilities of the b quark to hadronise into a B$_0$ or a B$_s$ meson, and only common systematic between LHCb and CMS), $f_d/f_s$, is factorised out.

Thus the full likelihood becomes

8 $$\begin{aligned}&L(BR_s,BR_d,f_d/f_s) \nonumber \\&\quad = L(f_d/f_s) \times L(BR_s,BR_d |f_d/f_s)_{LHCb} \nonumber \\&\qquad \times L(BR_s,BR_d | f_d/f_s)_{CMS} . \end{aligned}$$

where $BR_s$ and $BR_d$ stand for $\mathrm{BR}(B_s \rightarrow \mu ^+\mu ^-)$and $\mathrm{BR}(B_d \rightarrow \mu ^+\mu ^-)$, respectively. The log-likelihoods of quantities with asymmetric errors are approximated using a treatment equivalent to formula (4) in [115]. Equation 8 assumes the same value of $f_d/f_s$ for CMS and LHCb, which is the same assumption done in [108]. We then reparameterise the joint likelihood as a function of the single parameter of interest, $R^{exp}_{\mu \mu }$, imposing the constraint in (6) and using the SM prediction of $\mathrm{BR}(B_s \rightarrow \mu ^+\mu ^-)$ from [103–105].

Our final estimate after profiling on the theory uncertainties and $f_d/f_s$ is

9 $$\begin{aligned} R_{\mu \mu }^\mathrm{exp} = 0.92_{-0.20}^{+0.21}~. \end{aligned}$$

**Fig. 1:**
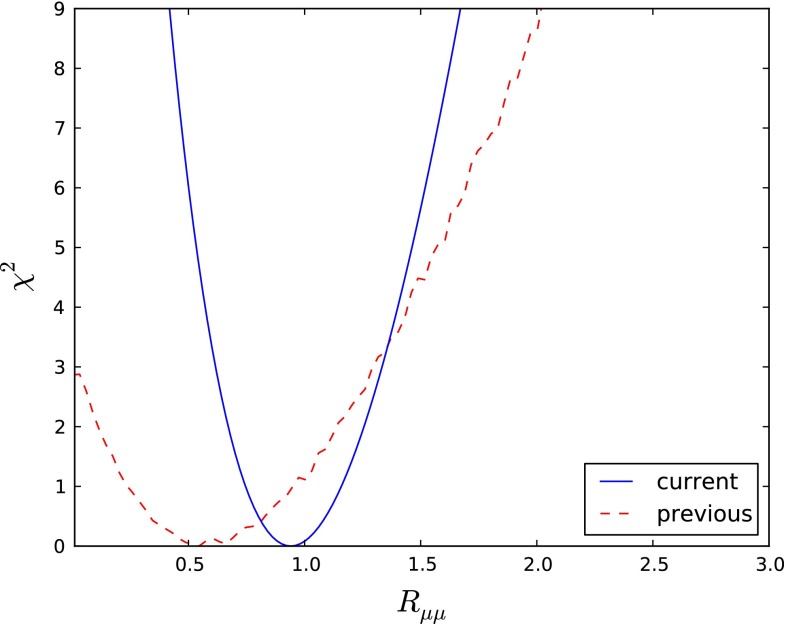
The contribution to the global $\chi ^2$ function of the LHCb and CMS measurements of $\mathrm{BR}(B_s \rightarrow \mu ^+\mu ^-)$ and $\mathrm{BR}(B_d \rightarrow \mu ^+\mu ^-)$ reported in (2) and (3), as calculated using the prescription described in the text (*blue solid line*) compared with the contribution calculated previously in [33] (*dashed red line*)

We have checked that our approach reproduces with good accuracy both the results in (5) when we use (8) and the $R_{\mu \mu }^\mathrm{CMS}$ value when we drop LHCb input from the final likelihood. Of course we performed both checks using the SM values for the branching ratios in [106, 107]. The contribution this function makes to the global $\chi ^2$ function is shown as the solid blue line in Fig. 1, where it is compared with the contribution calculated previously in [33] (dashed red line).

### The dark matter constraints

There are two important dark matter constraints on the CMSSM and NUHM1 parameter spaces. One is the cosmological dark matter density $\Omega _\chi h^2= 0.1198 \pm 0.0026$ estimated from Planck data [70], and the other is the upper limit on the spin-independent elastic cold dark matter scattering cross section $\sigma ^\mathrm{SI}_p$ from the LUX experiment [9], which is stronger by a factor $\sim$2 than that from the XENON100 experiment [8] in the range of neutralino masses relevant to this study. Upper limits on the spin-dependent cross section do not impinge on the parameter spaces of the models we study [30].

Previously, we used Micromegas 2.4.5 to calculate $\Omega _\chi h^2$, which we checked gave results similar to the independent SSARD code in the regions of interest. Here we use Micromegas 3.2 [85–87]. The recent results from the Planck satellite [70] refine the previous observational estimate of $\Omega _\chi h^2$, but this does not alter significantly the implications for other observables.

We compute the elastic scattering cross section, $\sigma ^\mathrm{SI}_p$, using [88]. There are important uncertainties in the calculation of $\sigma ^\mathrm{SI}_p$, which are now incorporated in the present analysis, which are also computed using [88]. There are two major sources for these uncertainties that we review here briefly. The first is the uncertainty related to the shift in the nucleon mass due to finite quark masses, $\sigma _0 = 36 \pm 7$ MeV, and the second is due to the uncertainty in the $\pi$–nucleon sigma term, $\Sigma _{\pi \!{\scriptscriptstyle N}}$, which we take here as $50 \pm 7$ MeV.

The spin-independent matrix element for $\tilde{\chi }^0_{1}$–nucleon scattering is proportional to a parameter $f_N$ that can be written as

10 $$\begin{aligned} \frac{f_N}{m_N} = \sum _{q={u},{d},{s}} f_{T_{q}}^{(N)} \frac{\alpha _{3q}}{m_{q}} + \frac{2}{27} f_{TG}^{(N)} \sum _{q={c},{b},{t}} \frac{\alpha _{3q}}{m_q} , \end{aligned}$$

where the parameters $f_{T_{q}}^{(N)}$ are defined by

11 $$\begin{aligned} m_N f_{T_{q}}^{(N)} \equiv \langle N | m_{q} \bar{q} q | N \rangle , \end{aligned}$$

with [116, 117]

12 $$\begin{aligned} f_{TG}^{(N)} = 1 - \sum _{q={u},{d},{s}} f_{T_{q}}^{(N)} . \end{aligned}$$

An expression for $\alpha _{3q}$ in terms of supersymmetric model parameters is given in [118]: it does not contribute significantly to the uncertainty in the calculation of the cross section, which is dominated by uncertainties in hadronic parameters.

These matrix elements are all directly proportional to $\Sigma _{\pi \!{\scriptscriptstyle N}}$. It is well known that the elastic cross section is very sensitive to the strange scalar density in the nucleon,

13 $$\begin{aligned} y = 1 - \sigma _0/\Sigma _{\pi \!{\scriptscriptstyle N}}\; . \end{aligned}$$

Indeed, $f_{T_s}$ is proportional to $\Sigma _{\pi \!{\scriptscriptstyle N}}y$, and hence the uncertainties in both $\Sigma _{\pi \!{\scriptscriptstyle N}}$ and $\sigma _0$ enter.

Our calculation of the uncertainty in the elastic cross section propagates the independent uncertainties in $$\Sigma _{\pi \!{\scriptscriptstyle N}}$$ and $$\sigma _0$$, as well as uncertainties in the quark-mass ratios $$m_\mathrm{d}/m_\mathrm{u}$$ and $$m_\mathrm{s}/m_\mathrm{d}$$, though the latter two are much smaller than the former two. For a more complete discussion of these uncertainties, see [119]. While the uncertainty in $$\sigma ^\mathrm{SI}_p$$ is often attributed to the uncertainty in $$\Sigma _{\pi \!{\scriptscriptstyle N}}$$, there is an almost equally large contribution to the uncertainty in $$\sigma ^\mathrm{SI}_p$$ coming from $$\sigma _0$$, particularly in the determination of the important strangeness contribution, $$ f_{T_{{s}}}$$.

**Fig. 2:**
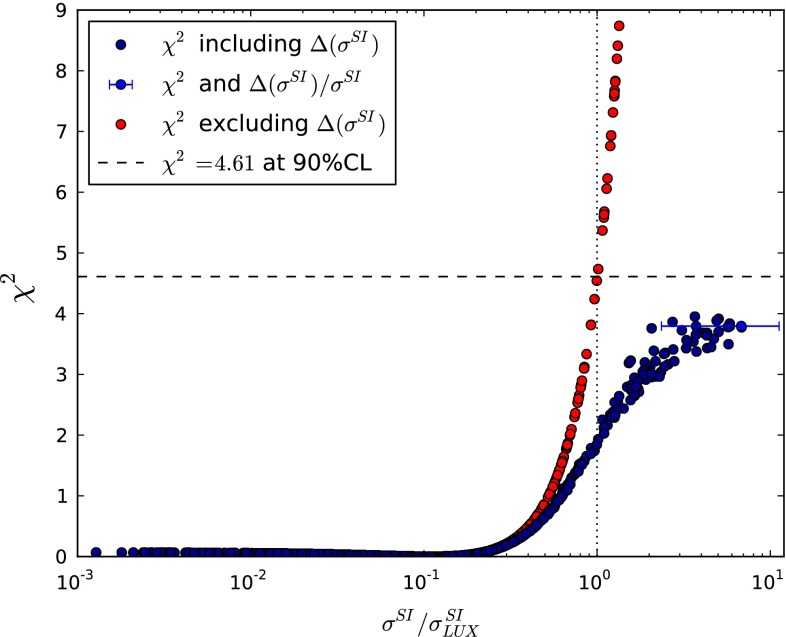
The contribution to the global $\chi ^2$ function that we calculate on the basis of the LUX 90 % CL upper limit on the spin-independent cross section $\sigma ^\mathrm{SI}_p$ [9], without (*red points*) and with (*blue points*) taking into account the theoretical uncertainty in the calculation of $\sigma ^\mathrm{SI}_p$. The *horizontal blue bar* exhibits the effect of this uncertainty in the hadronic matrix element on the calculation of $\sigma ^\mathrm{SI}_p$ for a specific CMSSM point

We display in Fig. 2 the contribution to the global $\chi ^2$ function that we calculate on the basis of the LUX 90 % CL upper limit on the spin-independent cross section $\sigma ^\mathrm{SI}_p$ [9], without (red points) and with (blue points) taking into account the uncertainty in the calculation of $\sigma ^\mathrm{SI}_p$. The horizontal blue bar is a representative example of the effect of the theoretical uncertainty in the hadronic matrix element on the calculation of $\sigma ^\mathrm{SI}_p$ for one of the CMSSM points.

## Results

### CMSSM fits

We now present the results of our new CMSSM fit using the above new inputs, considering first the case of $\mu > 0$ and then the case of $\mu < 0$. In each case, we present first some illustrative parameter planes, and then some one-dimensional likelihood functions.

#### Parameter planes for $\mu > 0$

**Fig. 3:**
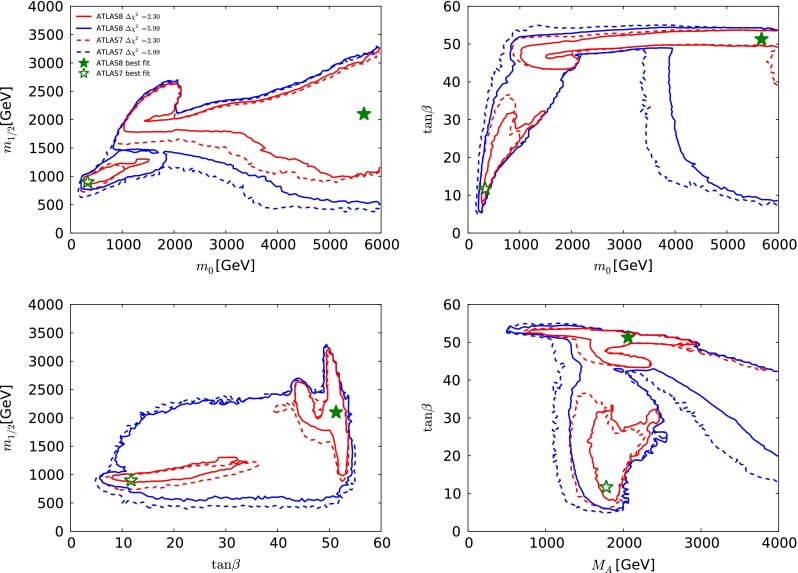
A compilation of parameter planes in the CMSSM for $\mu > 0$, including the $(m_0, m_{1/2})$ plane (*upper left*), the $(m_0, \tan \beta )$ plane (*upper right*), the $(\tan \beta , m_{1/2})$ plane (*lower left*), and the $(M_A, \tan \beta )$ plane (*lower right*), after implementing the ATLAS 20/fb jets + ${E\!\!/}_{T}$, $\mathrm{BR}(B_{s, d} \rightarrow \mu ^+\mu ^-)$, $M_h$, $\Omega _\chi h^2$, LUX constraints and other constraints as described in the text. The results of the current CMSSM fit are indicated by *solid lines* and *filled stars* and a fit to previous data [33] using the same implementations of the $M_h$, $\sigma ^\mathrm{SI}_p$ and other constraints is indicated by *dashed lines* and *open stars*. See the text for a detailed comparison of the current fit to that in [33]. The *red lines* denote $\Delta \chi ^2 = 2.30$ contours (corresponding approximately to the 68 % CL), and the *red lines* denote $\Delta \chi ^2 = 5.99$ (95 % CL) contours

In each of the parameter planes in Fig. 3, the best-fit point is indicated by a green star, the $\Delta \chi ^2 = 2.30$ contour that corresponds approximately to the 68 % CL is shown as a red line, and the $\Delta \chi ^2 = 5.99$ contour that corresponds approximately to the 95 % CL is shown as blue line. The results of the current fit are indicated by solid lines and solid stars. The results of fits to the same set of data constraints as used in our previous paper [33], but using current theoretical codes including FeynHiggs 2.10.0 and treating the dark matter scattering uncertainty as in Sect. 2.6, are shown as dashed lines and open stars. This comparison of the two results allows one to determine the effects of *new data*, independent of any code update. The effects of the new codes are discussed later.

We see in the upper left panel of Fig. 3 that the current CMSSM fit has two disjoint $\Delta \chi ^2 = 2.30$ contours in the $(m_0, m_{1/2})$ plane, one enclosing an ‘island’ at relatively low masses, centred around $(m_0, m_{1/2}) \sim (500, 1000) \,\, \mathrm {GeV}$, and a larger ‘continent’ extending from $(m_0, m_{1/2}) \sim (500, 1500) \,\, \mathrm {GeV}$ to larger mass values, beyond the range $m_0 < 6000 \,\, \mathrm {GeV}$ studied here. As we discuss below, the low-mass ‘island’ lies in the stau-coannihilation region, where the $(g-2)_\mu$ contribution to the global $\chi ^2$ function is reduced, whereas in the high-mass ‘continent’ the relic density is brought within the cosmological range by rapid $\tilde{\chi }^0_{1}$ annihilations due to direct-channel heavy Higgs boson resonances. Our current best-fit point lies in the outer 68 % CL region and has $(m_0, m_{1/2}) \sim (5650, 2100) \,\, \mathrm {GeV}$. There is a single $\Delta \chi ^2 = 5.99$ contour enclosing both the inner and the outer 68 % CL regions. We note that the global $\chi ^2$ function is quite flat in the outer region and very similar to the $\chi ^2$ value for the SM.^7^

The lower limit on $m_{1/2}$ at small $m_0 \sim 500 \,\, \mathrm {GeV}$ is provided mainly by the ATLAS 20/fb search for events with ${E\!\!/}_{T}$ and 2 to 6 jets, whereas at large $m_0 \gtrsim 3000 \,\, \mathrm {GeV}$ there are several relevant ATLAS limits using different event topologies with jets, leptons, $b$ quarks and ${E\!\!/}_{T}$. These are quite sensitive to $\tan \beta$ and $A_0$, and they have little impact on the preferred regions of the CMSSM parameter space, so we do not include them in our analysis. The lower limit on $m_0$ and the low-mass ‘island’ corresponds to the stau LSP boundary and the nearby coannihilation strip. The region at large $m_0$ and $m_{1/2}$ containing the best-fit point is in the rapid-annihilation funnel region, with the upper bound on $m_{1/2}$ being provided by the cosmological constraint on $\Omega _\chi h^2$. The region at small $m_{1/2}$ and large $m_0$ is in the focus-point region.

**Fig. 4:**
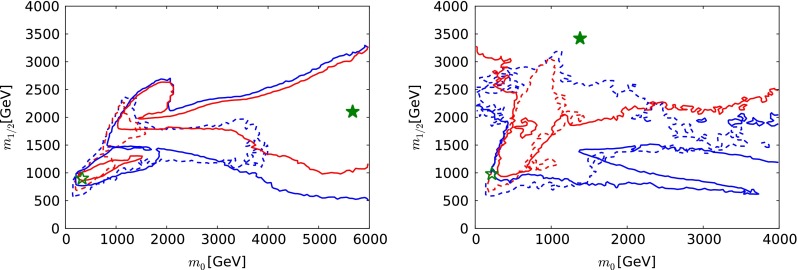
The $(m_0, m_{1/2})$ planes in the CMSSM (*left*) and the NUHM1 (right) for $\mu > 0$, comparing the results of the current CMSSM fit (*solid lines* and *filled stars*), with the results shown in [33] (*dashed lines* and *open stars*). The *red lines* denote $\Delta \chi ^2 = 2.30$ contours (corresponding approximately to the 68 % CL), and the *red lines* denote $\Delta \chi ^2 = 5.99$ (95 % CL) contours. See the text for a detailed comparison of the current fit to that in [33]

Looking now at the $(m_0, \tan \beta )$ plane in the upper right panel of Fig. 3, we see that the low-mass coannihilation ‘island’ corresponds to values of $\tan \beta \lesssim 30$, whereas the lower-$\chi ^2$ part of the high-mass continent corresponds to a band with larger values of $\tan \beta \sim 50$ in the rapid-annihilation funnel region, connected to a ‘continental shelf’ in the focus-point region extending to lower $\tan \beta$ when $m_0 \gtrsim 3500 \,\, \mathrm {GeV}$. The best-fit point has $\tan \beta \sim 50$ and lies in the funnel region.

The lower left panel of Fig. 3 shows the $(\tan \beta , m_{1/2})$ plane. As already mentioned, the only ATLAS 20/fb jets + ${E\!\!/}_{T}$ limit we use is that on ${E\!\!/}_{T}$ + 2 to 6 jets: other limits using topologies with leptons and/or $b$ jets could have an impact when $m_{1/2} \gtrsim 500 \,\, \mathrm {GeV}$, depending in particular on the value of $\tan \beta$ and/or $A_0$. We have not attempted to model these limits, but note that they would not affect the 68 % CL region displayed.

Finally, the lower right panel of Fig. 3 displays the $(M_A, \tan \beta )$ plane of the CMSSM. We see that in the low-mass coannihilation ‘island’ typical values of $M_A\sim 1500$ to 2500 GeV. The best-fit point has a similar value of $M_A$, but with a much larger value of $\tan \beta$. The band at large $\tan \beta$ corresponds to the rapid-annihilation funnel region. It is clear that the larger values of $m_0$ seen in the other panels correspond to large values of $M_A\sim 2500 \,\, \mathrm {GeV}$ and more.

We compare in Fig. 4 the results of the current analysis (solid lines and filled stars) with the results that were shown in [33] using the previous data set and the previous implementations of the constraints (dashed lines and open stars). As already mentioned, the strengthened ATLAS ${E\!\!/}_{T}$ constraints with 20/fb of data at 8 TeV have had little impact except to strengthen the lower limit on $m_{1/2}$ at low $m_0 \sim 500 \,\, \mathrm {GeV}$. At larger $m_0$, the range of $m_{1/2}$ values is broader than that shown in [33], largely because of changes in the MicrOMEGAs code between versions 2.4.5 and 3.2, which allow points with the correct dark matter relic density to be found in this region of parameter space. This is the case, in particular, for the best-fit point we now find, which lies in the outer 68 % CL region and has $(m_0, m_{1/2}) \sim (5650, 2100) \,\, \mathrm {GeV}$. Our new treatment of the uncertainty in $\sigma ^\mathrm{SI}_p$ discussed in Sect. 2.6, combined with larger Higgs mass found in FeynHiggs 2.10.0, has the effect of disfavouring the focus-point region [121] less than in [33], leading to an expansion in the region allowed at the 95 % CL at large $m_0$ and small $m_{1/2}$. The extension of the CMSSM 95 % CL region to larger $m_0$ in the left panel of Fig. 4 is due to the extended sampling range we use here: the MultiNest technique used here does not have a big impact beyond improving the density of sampling.

**Table 1:** The best-fit points found in global CMSSM fits for both signs of $\mu$ and an NUHM1 fit with $\mu > 0$, using the ATLAS 20/fb jets + ${E\!\!/}_{T}$ constraint [5], and the combination of the CMS [3] and LHCb [4] constraints on $\mathrm{BR}(B_{s, d} \rightarrow \mu ^+\mu ^-)$ [108], as well as an update of the FeynHiggs calculation of $M_h$ and a more conservative treatment of the hadronic matrix element uncertainties in $\sigma ^\mathrm{SI}_p$, as discussed in the text. The results for the CMSSM with $\mu > 0$ and the NUHM1 are compared with those found previously in global fits based on the ATLAS 7-TeV ${E\!\!/}_{T}$ data and the previous experimental constraint on $\mathrm{BR}(B_{s, d} \rightarrow \mu ^+\mu ^-)$, and with a current SM fit made using the procedure discussed in the text. We list the parameters of the best-fit points in both the low- and the high-mass ‘islands’ in Figs. 3, 8 and 12. The last column shows the fine-tuning for each point; see the text for more details. We note that the overall likelihood function is quite flat in both the CMSSM and the NUHM1, so that the precise locations of the best-fit points are not very significant, and we do not quote uncertainties. For completeness, we note that in the best NUHM1 fits $m_H^2 = - 2.54 \times 10^7 \,\, \mathrm {GeV}^2$ at the low-mass point and $m_H^2 \equiv = 1.33 \times 10^7 \,\, \mathrm {GeV}^2$ at the high-mass point

Model	Data set	Minimum $\chi ^2$/d.o.f.	Probability (%)	$m_0$ (GeV)	$m_{1/2}$ (GeV)	$A_0$ (GeV)	$\tan \beta$	Fine tuning
CMSSM	ATLAS 7 TeV	32.6/23	8.8	340	910	2670	12	800
$\mu > 0$	ATLAS$_\mathrm{20/fb}$ (low)	35.8/23	4.3	670	1040	3440	21	1200
	ATLAS$_\mathrm{20/fb}$ (high)	35.1/23	5.1	5650	2100	$-780$	51	1700
CMSSM	ATLAS$_\mathrm{20/fb}$ (low)	38.9/23	2.0	330	970	3070	10	1100
$\mu < 0$	ATLAS$_\mathrm{20/fb}$ (high)	36.6/23	3.6	6650	2550	$-3150$	39	1900
NUHM1	ATLAS 7 TeV	30.5/22	10.7	370	1120	5130	8	6000
$\mu > 0$	ATLAS$_\mathrm{20/fb}$ (low)	33.3/22	5.8	470	1270	5700	11	10000
	ATLAS$_\mathrm{20/fb}$ (high)	32.7/22	6.6	1380	3420	$-3140$	39	2000
“SM”	ATLAS$_\mathrm{20/fb}$ (high)	36.5/24	5.0	–	–	–	–	–

The global likelihood function calculated in [33] had two local minima with almost equal values of $\chi ^2$. The new ATLAS constraint and the new implementations of the $M_h$ and $\sigma ^\mathrm{SI}_p$ constraints combine to slightly disfavour the local minimum in the low-mass ‘island’ by only $\sim 0.7$ in $\chi ^2$ compared to the global minimum at $(m_0, m_{1/2}) \sim (5650, 2100) \,\, \mathrm {GeV}$, where the main contribution comes from the ATLAS ${E\!\!/}_{T}$ constraint. As in the case of our previous analysis [33], the $\mathrm{BR}(B_{s, d} \rightarrow \mu ^+\mu ^-)$ constraint does not play a large rôle in the current fit. Its main importance is at large $\tan \beta$ and small $m_0, m_{1/2}$ and $M_A$, but the low-mass ‘island’ has small $\tan \beta$ and the current best-fit point has large values of $m_0, m_{1/2}$ and $M_A$.

#### Characteristics of the best-fit points for $\mu > 0$

Table 1 summarises the values of $\chi ^2$ and the locations of the best-fit points found in the current analysis in the low-mass (‘island’) and high-mass (‘continent’) regions of the CMSSM parameter space with $\mu > 0$.^8^ We see that although the minimum value of $\chi ^2$ in the ‘continent’ is smaller than in the ‘island’, the difference is less than unity and hence is not significant. As already mentioned, the ‘island’ best-fit point is in the stau-coannihilation region, whereas rapid annihilation via direct-channel $H/A$ poles is dominant at the best-fit point in the high-mass ‘continent’.

Comparing with the best fit found previously in the CMSSM using the ATLAS 7-TeV ${E\!\!/}_{T}$ constraint and the previous $\mathrm{BR}(B_{s, d} \rightarrow \mu ^+\mu ^-)$ measurement, we see that the best-fit $\chi ^2$ has increased by about 2.1 and the ‘island’ $\chi ^2$ by about 3. Thus the pressure exerted by the ATLAS 20/fb jets + ${E\!\!/}_{T}$ and $\mathrm{BR}(B_{s, d} \rightarrow \mu ^+\mu ^-)$ constraints does not change significantly the overall picture for the CMSSM. Specifically, the values of $m_0$ and $m_{1/2}$ at the new best-fit ‘island’ point are not very different from those at the previous CMSSM best-fit point, though the values of $A_0$ and $\tan \beta$ have changed substantially. Also shown for comparison is the value of $\chi ^2$ for the “SM”, as calculated using the MasterCode by setting $m_0 = m_{1/2} = 15$ TeV. We see that the CMSSM is unable to reduce $\chi ^2$ much below the “SM” value, with a similar fit probability.

The last column in Table 1 lists the amounts of fine-tuning of SUSY parameters required at each of the best-fit points, as calculated using the measure suggested in [127, 128]. The CMSSM and NUHM1 low-mass best-fit points do not require significantly more fine-tuning than those found in our previous analysis [33], whereas the high-mass points do. However, we caution against using this as a strong argument in favour of the low-mass points, in view of the subjective nature of the fine-tuning measure and the ambibuities in its interpretation.

**Table 2:** Summary of the contributions of the most relevant observables to the global $\chi ^2$ function at the best-fit high- and low-mass points in the CMSSM (with both signs of $\mu$) and NUHM1 (with $\mu > 0$), including the recently updated observables ATLAS 20/fb jets + ${E\!\!/}_{T}$, $\mathrm{BR}(B_{s, d} \rightarrow \mu ^+\mu ^-)$ and the LUX upper limit on dark matter scattering. As noted in parentheses, within the SM, $\Delta \chi ^2 \sim 1.5$ is found in [73] due to the (small) tension between the measured value of $M_h$ and the precision electroweak data

Observable	$\Delta \chi ^2$ CMSSM $\mu > 0$ (high)	$\Delta \chi ^2$ CMSSM $\mu > 0$ (low)	$\Delta \chi ^2$ CMSSM $\mu < 0$ (high)	$\Delta \chi ^2$ CMSSM $\mu < 0$ (low)	$\Delta \chi ^2$ NUHM1 $\mu > 0$ (high)	$\Delta \chi ^2$ NUHM1 $\mu > 0$ (low)	$\Delta \chi ^2$ Standard model
Global	35.1	35.8	36.6	38.9	32.7	33.3	36.5
BR$_\mathrm{b \rightarrow s \gamma }^\mathrm{exp/SM}$	0.52	1.58	0.37	0.00	0.54	0.02	0.57
BR$_\mathrm{B \rightarrow \tau \nu }^\mathrm{exp/SM}$	1.77	1.63	1.63	1.61	1.65	1.66	1.60
$\epsilon _K$	1.94	1.88	1.94	1.87	1.94	1.94	1.96
$a_{\mu }^\mathrm{exp} - a_{\mu }^\mathrm{SM}$	10.71	9.34	11.42	12.65	10.50	9.63	11.19
$M_W$	1.35	0.22	2.15	0.04	0.00	0.11	1.38
$M_h$	0.00	0.04	0.03	0.53	0.00	0.22	(1.5)
$R_\ell$	1.10	1.04	1.10	1.00	1.07	1.00	1.09
$A_\mathrm{fb}({b})$	6.56	6.79	6.05	7.61	5.45	6.93	6.58
$A_\ell (\mathrm{SLD})$	3.59	3.40	3.99	2.81	4.59	3.30	3.55
$\sigma _\mathrm{had}^0$	2.52	2.55	2.56	2.51	2.59	2.56	2.54
LUX	0.03	0.07	0.66	0.07	0.00	0.07	–
ATLAS 20/fb	0.04	2.52	0.02	3.35	0.02	1.15	–
$B_{s,d} \rightarrow \mu ^+ \mu ^-$	0.51	0.46	0.13	0.11	0.22	0.35	0.15

Table 2 gives more details of the contributions to the global $\chi ^2$ function from different observables in the CMSSM at the high- and low-mass best-fit points, compared with our implementation of the SM. At both the high- and the low-mass points, the $M_h$ measurement makes a small contribution to the global $\chi ^2$ function. We see that the low-mass point has less tension with $(g-2)_\mu$, and it is favoured by both $M_W$ and $\mathrm{BR}(B_{s,d}\rightarrow \mu ^+\mu ^-)$, in particular, whereas the high-mass point is preferred by $\mathrm{BR}(b\rightarrow s\gamma )$ and ATLAS 20/fb jets + ${E\!\!/}_{T}$, in particular. The “SM” fit is noticeably worse for $(g-2)_\mu$ and $M_W$.

#### One-dimensional likelihood functions for $\mu > 0$

**Fig. 5:**
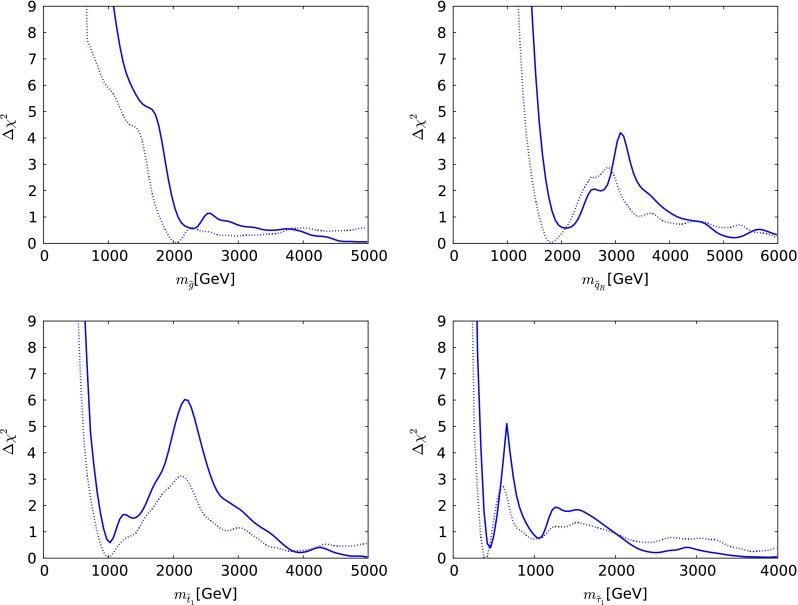
The one-dimensional $\chi ^2$ likelihood functions in the CMSSM for $\mu > 0$ for $m_{\tilde{g}}$ (*upper left*), $m_{\tilde{q}_R}$ (*upper right*), $m_{\tilde{t}_1}$ (*lower left*) and $m_{\tilde{\tau }_1}$ (*lower right*). In each panel, the *solid line* is derived from a global analysis of the present data, and the *dotted line* is obtained from a reanalysis of the data used in [33], using the implementations of the $M_h$ and $\sigma ^\mathrm{SI}_p$ constraints discussed in Sect. 2

We now present the one-dimensional $\chi ^2$ likelihood functions for various particle masses and other observables when $\mu > 0$, which are shown as continuous lines in Fig. 5 (the dotted lines are discussed below). The upper left panel displays the $\chi ^2$ function for $m_{\tilde{g}}$. We see that it falls essentially monotonically for $m_{\tilde{g}}\gtrsim 1000 \,\, \mathrm {GeV}$, a feature that masks the structures seen in the upper left panel of Fig. 3. The one-dimensional projection merges the low-mass ‘island’ and the high-mass ‘continent’ that are separated in the $(m_0, m_{1/2})$ plane of the CMSSM. It is to be expected that the $\chi ^2$ function continues close to zero also at larger values of $m_{\tilde{g}}$.

**Fig. 6:**
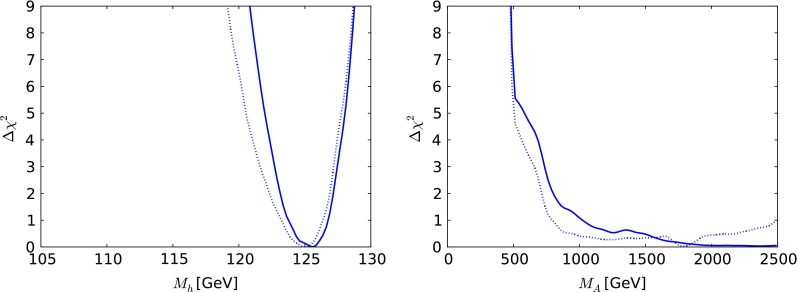
The one-dimensional $\chi ^2$ likelihood functions in the CMSSM for $\mu > 0$ for $M_h$ (*left*) and $M_A$ (*right*). In each panel, the *solid line* is derived from a global analysis of the present data, and the *dotted line* is derived from a reanalysis of the data used in [33], using the implementations of the $M_h$ and $\sigma ^\mathrm{SI}_p$ constraints discussed in Sect. 2

The $\chi ^2$ function for $m_{\tilde{q}_R}$ seen in the upper right panel of Fig. 5 exhibits more structure, with a local minimum at $m_{\tilde{q}_R}\sim 2200 \,\, \mathrm {GeV}$, a local maximum at $m_{\tilde{q}_R}\sim 3000 \,\, \mathrm {GeV}$, and then an essentially monotonic fall at larger $m_{\tilde{q}_R}$. The appearance of the local minimum can be understood by remembering that $m_{\tilde{q}_R}^2 \sim m_0^2 + 5 m_{1/2}^2$, so that the value of $m_{\tilde{q}_R}$ is fixed along elliptical contours in the $(m_0, m_{1/2})$ plane. The local minimum in the $\chi ^2$ function for $m_{\tilde{q}_R}$ corresponds to an ellipse passing through the red ‘island’ in the upper left panel of Fig. 3, and the local maximum corresponds to an ellipse passing between the ‘island’ and the ‘continent’. However, we should emphasise that neither the local minimum nor the local maximum is very significant, since they have $\Delta \chi ^2 \sim 1, 4$ relative to the minimum value of $\chi ^2$.

Similar features are seen in the $\chi ^2$ function for the mass of the lighter stop squark, $m_{\tilde{t}_1}$, as seen in the lower left panel of Fig. 5. However, in this case the local minimum appears at a lower mass $m_{\tilde{t}_1}\sim 1000 \,\, \mathrm {GeV}$, and the local maximum is also at a lower mass $m_{\tilde{t}_1}\sim 2000 \,\, \mathrm {GeV}$, reflecting the fact that the isomass contours for $m_{\tilde{t}_1}$ and $m_{\tilde{q}_R}$ are different. As in many other models, we find that the ${\tilde{t}_1}$ is likely to be considerably lighter than the other strongly interacting sparticles. This is due to a large mixing in the scalar top sector, driven by the relatively large value of $M_h$.

Similar local structures can also be seen in the $\chi ^2$ function for the lighter stau, $m_{\tilde{\tau }_1}$, as seen in the lower right panel of Fig. 5. In this case, the local minimum is at $m_{\tilde{\tau }_1}\sim 450 \,\, \mathrm {GeV}$, nearly degenerate with the lightest neutralino, and placing the ${\tilde{\tau }_1}$ and other sleptons beyond the reach of an $e^+ e^-$ collider with $E_\mathrm{CM} \lesssim 900 \,\, \mathrm {GeV}$. We also find that $\Delta \chi ^2 > 9$ for $m_{\tilde{\tau }_1}< 300 \,\, \mathrm {GeV}$. We also see a second local minimum of $\chi ^2$ at $m_{\tilde{\tau }_1}\sim 1000 \,\, \mathrm {GeV}$, which arises from the lobe at $(m_0, m_{1/2}) \sim (1500, 2200)$ in the upper left panel of Fig. 3. However, we emphasise that these observations are very model-dependent.

We now comment briefly on the differences between the one-dimensional likelihood functions found in our analysis of the current data, and those found using the same implementations of the $M_h$ and $\sigma ^\mathrm{SI}_p$ constraints for the data set used in [33], shown in Fig. 5 as dotted lines. The current likelihood functions for $m_{\tilde{g}}, m_{\tilde{q}_R}, m_{\tilde{t}_1}$ and $m_{\tilde{\tau }_1}$ are generally higher at small masses, where the ATLAS ${E\!\!/}_{T}$ search has the most impact, but are similar at high masses.

Figure 6 displays the $\chi ^2$ functions for the mass of the lightest SUSY Higgs boson, $M_h$, shown in the left panel and the mass of the pseudoscalar Higgs boson, $M_A$, shown in the right panel. We see that the likelihood for $M_h$ is well maximised close to the measured Higgs mass. The likelihood for $M_A$ is very flat for $M_A\gtrsim 1000 \,\, \mathrm {GeV}$, with $\Delta \chi ^2$ rising rapidly to reach $> 9$ for $M_A< 500 \,\, \mathrm {GeV}$, and it is very similar to the likelihood found using the same data set as in [33].

On the basis of these one-dimensional likelihood functions we can establish 95 % CL lower limits on $m_{\tilde{g}}, m_{\tilde{q}_R}, m_{\tilde{t}_1}$ and $m_{\tilde{\tau }_1}$ for the CMSSM with $\mu > 0$, which are listed in the second column of Table 3. Reflecting the relatively large values of $m_0$ favoured in this analysis, we see that the lower limit on $m_{\tilde{q}_R}$ is considerably stronger than that on $m_{\tilde{g}}$. On the other hand, the ${\tilde{t}_1}$ could be substantially lighter than the other strongly interacting sparticles.

**Table 3:** The 95 % CL lower limits (in GeV) on various sparticle masses in the CMSSM with both signs of $\mu$ and the NUHM1 with $\mu > 0$. We emphasise that these limits are specific to the models studied. In the case of the CMSSM with $\mu < 0$ and the NUHM1, the parentheses indicate the approximate locations of small mass ranges where the $\chi ^2$ function dips briefly below the 95 % CL

Sparticle	CMSSM	CMSSM	NUHM
	$\mu > 0$	$\mu < 0$	$\mu > 0$
${\tilde{g}}$	1810	(2100) (3200) 3540	1920
${\tilde{q}_R}$	1620	(1900) 6300	1710
${\tilde{t}_1}$	750	(950) 4100	(650) 1120
$\tilde{\tau }_1$	340	(400) 4930	380
$M_A$	690	(1900) 3930	450

**Fig. 7:**
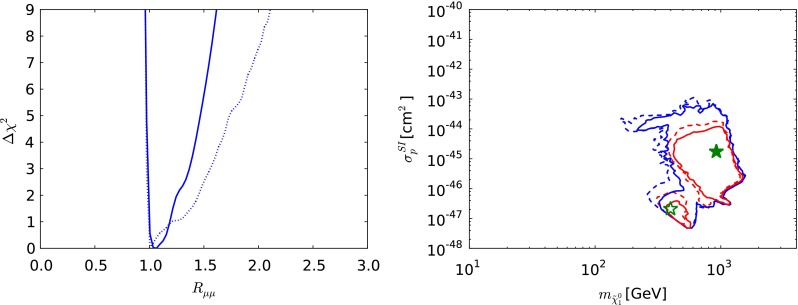
The one-dimensional $\chi ^2$ likelihood function in the CMSSM for $\mu > 0$ for $\mathrm{BR}(B_{s, d} \rightarrow \mu ^+\mu ^-)$ (*left*) and the $(m_{\tilde{\chi }^0_{1}} , \sigma ^\mathrm{SI}_p)$ plane (*right*). In both panels, the *solid lines* are derived from a global analysis of the present data and the *dotted lines* are derived from a reanalysis of the data used in [33], using the implementations of the $M_h$ and $\sigma ^\mathrm{SI}_p$ constraints discussed in Sect. 2. In the *right panel*, the *red lines* denote the $\Delta \chi ^2 = 2.30$ contours, the *blue lines* denote the $\Delta \chi ^2 = 5.99$ contours in each case and the filled (*open*) *green star* denotes the corresponding best-fit point

The left panel of Fig. 7 displays the likelihood function for $\mathrm{BR}(B_{s, d} \rightarrow \mu ^+\mu ^-)$, which is seen to be minimised close to the SM value. The rise at larger $\mathrm{BR}(B_{s, d} \rightarrow \mu ^+\mu ^-)$ is largely due to the direct experimental constraint on this quantity, but the steep rise at lower $\mathrm{BR}(B_{s, d} \rightarrow \mu ^+\mu ^-)$ is due to the other constraints on the CMSSM, which are hard to reconcile with $R_{\mu \mu } < 1$. The rise at large $\mathrm{BR}(B_{s, d} \rightarrow \mu ^+\mu ^-)$ found from the data set used in [33] is less steep, reflecting the evolution in the measurement of $\mathrm{BR}(B_{s, d} \rightarrow \mu ^+\mu ^-)$. The right panel of Fig. 7 displays the $(m_{\tilde{\chi }^0_{1}} , \sigma ^\mathrm{SI}_p)$ plane, again with solid (dashed) lines representing the current analysis (the constraints of [33]), respectively, with the filled (open) green star denoting the corresponding best-fit point whereas the red (blue) lines representing 68 (95) % CL contours, respectively. We see that a range $10^{-47}\,\mathrm{cm}^2 \lesssim \sigma ^\mathrm{SI}_p\ \lesssim 10^{-43}$ cm$^2$ is allowed at the 95 % CL, and the best-fit point yields a value in the middle part of this range $\sim 10^{-45}$ cm$^2$. The mass of $m_{\tilde{\chi }^0_{1}}$ at the best-fit point is $935 \,\, \mathrm {GeV}$. Since the favoured range of $m_{\tilde{\chi }^0_{1}}$ is high, in this and the other models discussed later, and the predicted values of $\sigma ^\mathrm{SI}_p$ correspondingly small, the search for spin-independent dark matter scattering does not have a strong impact on the global fits.

**Fig. 8:**
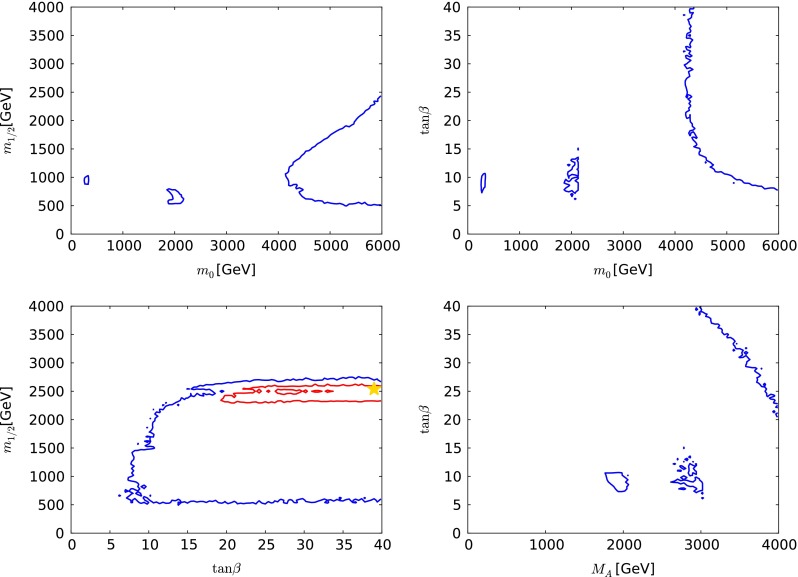
As in Fig. 3, but for $\mu < 0$ in the CMSSM. For the reason discussed in the text, only the ranges $\tan \beta \le 40$ are displayed. The *yellow star* in the *lower left panel* marks the best-fit point in the CMSSM with $\mu < 0$, which is out of the ranges of the other panels

#### Comparisons between analyses

We restrict our attention here to the only other analysis that incorporates the latest ATLAS 20/fb jets + ${E\!\!/}_{T}$ constraint. Preliminary results from a new global frequentist analysis of the CMSSM with $\mu > 0$ within the FITTINO framework have recently been presented [47]. The best-fit point found in [47] is very similar to the best-fit point we find in the low-mass region of the CMSSM with $\mu > 0$. However, the regions of the parameter space favoured at the 68 and 95 % CL in the FITTINO analysis do not extend to values of $(m_0, m_{1/2})$ as large as those we find in the present analysis. In addition to ATLAS 20/fb jets + ${E\!\!/}_{T}$, this analysis also uses HIGGSSIGNALS [120] to derive constraints from the Higgs-mass and signal-strength measurements. The latter do not change substantially the results, since the Higgs rate predictions in the favoured regions of the CMSSM parameter space, which are in the in the decoupling regime,^9^ are quite similar to those in the SM and do not vary significantly.^10^

### CMSSM with $\mu < 0$

The case $\mu < 0$ has been studied less than $\mu > 0$ (but see, e.g., [65, 66, 124, 125]), for various reasons: It *worsens* the discrepancy between the experimental value of $(g-2)_\mu$ and the SM calculation, it is in general *more restricted* by $\mathrm{BR}(b \rightarrow s \gamma )$  and it yields a *smaller* value of $M_h$ for fixed values of the other CMSSM parameters. However, since the ATLAS 20/fb jets + ${E\!\!/}_{T}$ and other constraints require relatively large values of $m_0$ and $m_{1/2}$ where the SUSY contribution to $(g-2)_\mu$ and $\mathrm{BR}(b \rightarrow s \gamma )$ are small, it is appropriate to reconsider the $\mu < 0$ case.

**Fig. 9:**
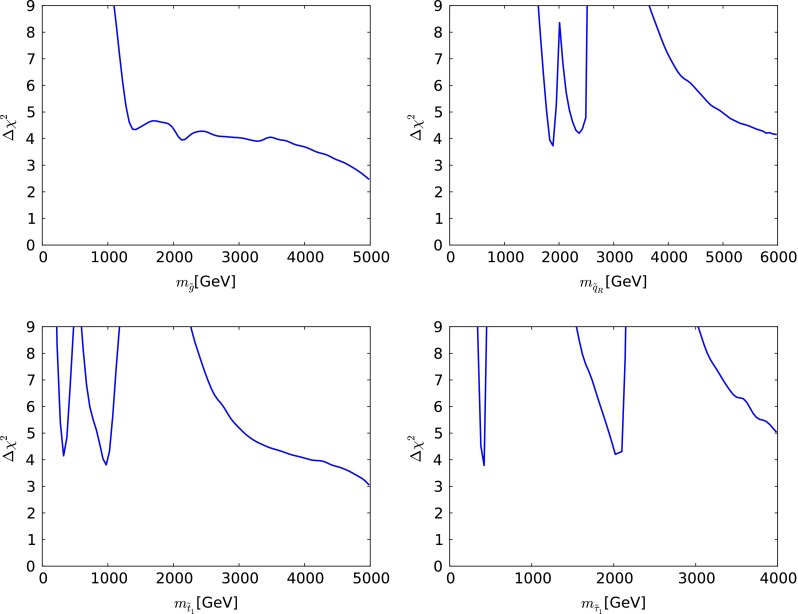
The one-dimensional $\chi ^2$ likelihood functions in the CMSSM for $\mu < 0$ for $m_{\tilde{g}}$ (*upper left*), $m_{\tilde{q}_R}$ (*upper right*), $m_{\tilde{t}_1}$ (*lower left*) and $m_{\tilde{\tau }_1}$ (*lower right*). In each panel, the *solid line* is derived from a global analysis of the present data using the implementations of the $M_h$ and $\sigma ^\mathrm{SI}_p$ constraints discussed in Sect. 2

#### Parameter planes with $\mu < 0$

We see in the upper left panel of Fig. 8 that there are three regions of the $(m_0, m_{1/2})$ plane that are allowed at the 95 % level, two small ‘reefs’ at relatively low masses $(m_0, m_{1/2}) \sim (300, 1000)$ and $(600, 2000) \,\, \mathrm {GeV}$ and a more extensive ‘continent’ at larger masses $m_0 \gtrsim 4000 \,\, \mathrm {GeV}$. The lower-mass ‘reef’ is in the stau-connihilation region, as in the $\mu > 0$ case, but the higher-mass ‘reef’ is in the stop-coannihilation region. Compared to the high-mass ‘continent’ in the rapid-annihilation funnel and focus-point regions, the ‘reef’ has smaller contributions to the global $\chi ^2$ function for some electroweak and flavour observables, but is disfavoured by ATLAS 20/fb jets + ${E\!\!/}_{T}$. The best-fit point in the CMSSM for $\mu < 0$ is shown as a yellow star: it is located in the high-mass ‘continent’, in the focus-point region.

The $(m_0, \tan \beta )$ plane for $\mu < 0$ is shown in the upper right panel of Fig. 8.^11^ Here we see that the low-mass ‘reefs’ are restricted to $5 \lesssim \tan \beta \lesssim 15$, whereas the ‘continent’ extends over all $\tan \beta \gtrsim 8$. In the lower left panel of Fig. 8, we see in the $(\tan \beta , m_{1/2})$ plane that the ‘reefs’ and ‘continent’ merge in this projection of the CMSSM parameter space with $\mu < 0$. We also see that $500~\,\, \mathrm {GeV}\lesssim m_{1/2} \lesssim 2500 \,\, \mathrm {GeV}$ is allowed at the 95 % CL for the range $m_0 < 6000 \,\, \mathrm {GeV}$ studied here.^12^ The small region within the red 68 % contour does not appear in the other panels, because it corresponds to values of $m_0 > 6000 \,\, \mathrm {GeV}$ and $M_A> 4000 \,\, \mathrm {GeV}$, which are not displayed in the other panels of Fig. 8. Finally, in the lower right panel of Fig. 8 we see in the $(M_A, \tan \beta )$ plane that only in the ‘reefs’ are values of $M_A\lesssim 3000 \,\, \mathrm {GeV}$ are allowed at the 95 % CL when $\tan \beta \le 40$.^13^ The ‘reef’s are again clearly separated at relatively small values of $\tan \beta$, with a restricted range of $M_A\in (2000, 3000) \,\, \mathrm {GeV}$.

#### Characteristics of the best-fit points for $\mu < 0$

We display in Table 1 the characteristics of the best-fit points in the CMSSM with $\mu < 0$ in the low-mass ‘reef’ region and the high-mass ‘continent’. Unlike the case of the CMSSM with $\mu > 0$, ${\tilde{t}_1}$ coannihilation is important at the best-fit point in the ‘reef’ region, and ${\tilde{\chi }^\pm _{1}}, \tilde{\chi }^0_{2}, \tilde{\chi }^0_{3}$ coannihilation at the best-fit ‘continental’ point. In both cases, the global $\chi ^2$ function is somewhat higher than in the corresponding regions for $\mu > 0$, by $\sim 3.1$ in the low-mass region and by $\sim 1.5$ in the high-mass region. The main origins of the differences can be seen in Table 2. The high-mass model with $\mu < 0$ receives larger contributions from $(g-2)_\mu$, $M_W$ and $\sigma ^\mathrm{SI}_p$, whereas there are larger contributions from $(g-2)_\mu$ and $M_h$ in the low-mass case, compensated only partially by smaller $\chi ^2$ contributions from $\mathrm{BR}(b \rightarrow s \gamma )$ and $\mathrm{BR}(B_{s, d} \rightarrow \mu ^+\mu ^-)$. As a result, the best-fit CMSSM points with $\mu < 0$ have higher $\chi ^2$ and lower fit probabilities than the SM.

#### One-dimensional likelihood functions for $\mu < 0$

We display in Fig. 9 the one-dimensional $\chi ^2$ functions for various sparticle masses in the CMSSM with $\mu < 0$. We see in the upper left panel that the $\chi ^2$ function for $m_{\tilde{g}}$ falls essentially monotonically as $m_{\tilde{g}}\rightarrow 5000 \,\, \mathrm {GeV}$ towards $\Delta \chi ^2 \sim 2.5$ relative to the global minimum. The best fit for $\mu < 0$ has $\Delta \chi ^2 \sim 1.8$ at $m_{\tilde{g}}\sim 5300 \,\, \mathrm {GeV}$, and hence it is not seen in this plot.

On the other hand, the one-dimensional $\chi ^2$ function for $m_{\tilde{q}_R}$, shown in the upper right panel of Fig. 9 has a very different form. After falling initially to $\Delta \chi ^2 \sim 4$, there is a local maximum at $m_{\tilde{q}_R}\sim 2000 \,\, \mathrm {GeV}$ with $\Delta \chi ^2 \sim 8$. This is followed by a region where $\Delta \chi ^2$ falls again to $\sim 4$, followed by a sharp rise to $\Delta \chi ^2 > 9$. Finally, the $\chi ^2$ function falls again below $\Delta \chi ^2 = 9$ when $m_{\tilde{q}_R}> 3800 \,\, \mathrm {GeV}$ and continues falling with increasing $m_{\tilde{q}_R}$. The low-mass structures are in the ‘reef’ regions and the high-mass fall is in the ‘continental’ region. Similar features are seen in the $\chi ^2$ function for $m_{\tilde{t}_1}$, but at lower masses, in the lower left panel of Fig. 9. The $\chi ^2$ function for $m_{\tilde{\tau }_1}$ shown in the lower right panel of Fig. 9 exhibits sharp local minima at $m_{\tilde{\tau }_1}\sim 400$ (associated with the dip in the gluino $\chi ^2$ at $2000$ GeV) and $2000 \,\, \mathrm {GeV}$ (associated with the high gluino-mass region), followed again by a decrease across the ‘continent’ at large masses.

**Fig. 10:**
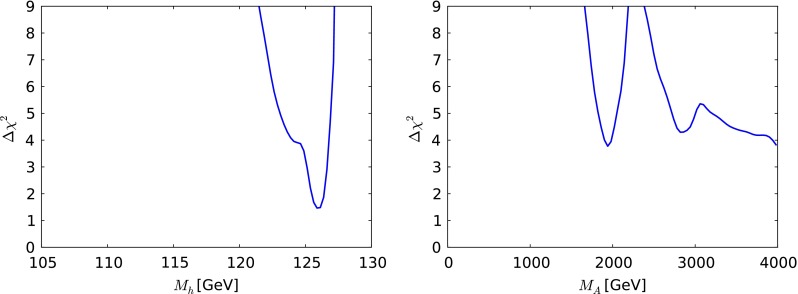
The one-dimensional $\chi ^2$ likelihood functions in the CMSSM for $\mu < 0$ for $M_h$ (*left*) and $M_A$ (*right*). In each panel, the *solid line* is derived from a global analysis of the using the implementations of the $M_h$ and $\sigma ^\mathrm{SI}_p$ constraints discussed in Sect. 2

We display in Fig. 10 the one-dimensional $\chi ^2$ functions for $M_h$ (left panel) and $M_A$ (right panel) as calculated using FeynHiggs 2.10.0. We see that $M_h$ has a well-defined minimum at $M_h\sim 126 \,\, \mathrm {GeV}$. The fact that low values of $M_h\lesssim 122 \,\, \mathrm {GeV}$ do not acquire a heavier $\chi ^2$ penalty is due to the theoretical uncertainty in the calculation of $M_h$, which we take to be $1.5 \,\, \mathrm {GeV}$. The $\chi ^2$ function for $M_A$ has a local minimum at $M_A\sim 2000 \,\, \mathrm {GeV}$ followed by a rise to a local maximum at $M_A\sim 2300 \,\, \mathrm {GeV}$ and then a decrease towards $\Delta \chi ^2 \sim 4$ when $M_A\sim 4000 \,\, \mathrm {GeV}$.

**Fig. 11:**
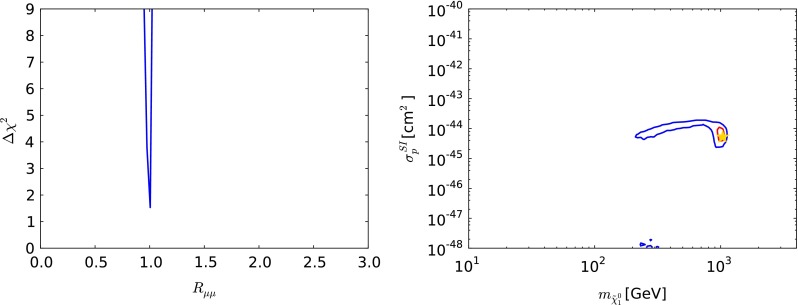
The one-dimensional $\chi ^2$ likelihood function in the CMSSM for $\mu < 0$ for $\mathrm{BR}(B_{s, d} \rightarrow \mu ^+\mu ^-)$ (*left*) and the $(m_{\tilde{\chi }^0_{1}} , \sigma ^\mathrm{SI}_p)$ plane (*right*). In both panels, the *solid lines* are derived from a global analysis of the present data using the implementations of the $M_h$ and $\sigma ^\mathrm{SI}_p$ constraints discussed in Sect. 2. In the *right panel*, the *red lines* denote the $\Delta \chi ^2 = 2.30$ contours, the *blue lines* denote the $\Delta \chi ^2 = 5.99$ contours in each case and the *filled yellow star* denotes the corresponding best-fit point

**Fig. 12:**
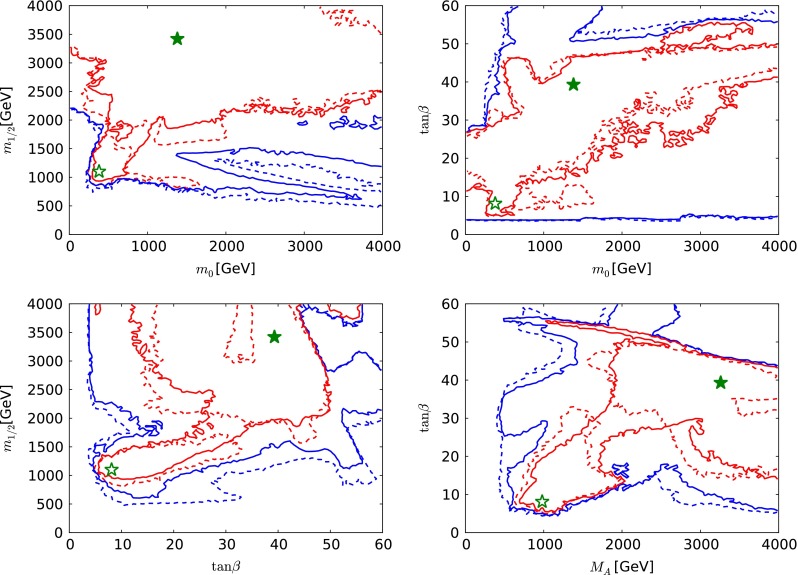
A compilation of parameter planes in the NUHM1 for $\mu > 0$, including the $(m_0, m_{1/2})$ plane (*upper left*), the $(m_0, \tan \beta )$ plane (*upper right*), the $(\tan \beta , m_{1/2})$ plane (*lower left*), and the $(M_A, \tan \beta )$ plane (*lower right*), after implementing the ATLAS 20/fb jets + ${E\!\!/}_{T}$, $\mathrm{BR}(B_{s, d} \rightarrow \mu ^+\mu ^-)$, $M_h$, $\Omega _\chi h^2$, LUX constraints and other constraints as described in the text. The results of the current NUHM1 fit are indicated by *solid lines* and *filled stars*, and a fit to previous data [33] using the same implementations of the $M_h$, $\sigma ^\mathrm{SI}_p$ and other constraints is indicated by *dashed lines* and *open stars*. See the text for a detailed comparison of the current fit to that in [33]. The *red lines* denote $\Delta \chi ^2 = 2.30$ contours (corresponding approximately to the 68 % CL), and the red lines denote $\Delta \chi ^2 = 5.99$ (95 % CL) contours

**Fig. 13:**
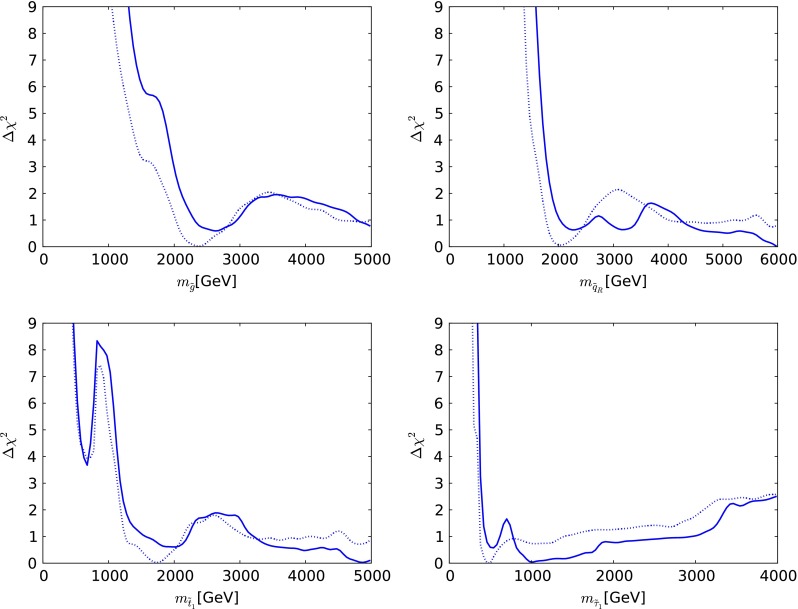
The one-dimensional $\chi ^2$ likelihood functions in the NUHM1 for $\mu > 0$ for $m_{\tilde{g}}$ (*upper left*), $m_{\tilde{q}_R}$ (*upper right*), $m_{\tilde{t}_1}$ (*lower left*) and $m_{\tilde{\tau }_1}$ (*lower right*). In each panel, the *solid line* is derived from a global analysis of the present data, and the *dotted line* is obtained from a reanalysis of the data used in [33], using the implementations of the $M_h$ and $\sigma ^\mathrm{SI}_p$ constraints discussed in Sect. 2

These one-dimensional likelihood functions can be used to set 95 % lower limits on various sparticle masses by requiring $\Delta \chi ^2 < 4$ relative to the global minimum for the CMSSM, which occurs for $\mu > 0$ as discussed earlier. These lower limits are tabulated in the third column of Table 3. We indicate in parentheses the approximate locations of limited ranges of masses where the $\chi ^2$ function dips briefly below the 95 % CL.

Figure 11 shows the one-dimensional $\chi ^2$ functions for $\mathrm{BR}(B_{s, d} \rightarrow \mu ^+\mu ^-)$ (left panel) and $\sigma ^\mathrm{SI}_p$ (right panel) for $\mu < 0$. We see that $\mathrm{BR}(B_{s, d} \rightarrow \mu ^+\mu ^-)$ is expected to be very similar to the SM value, reflecting the previous observation that the lowest $\chi ^2$ values for $\mu > 0$ are attained in the ‘continent’ at large sparticle masses and large $M_A$, and the secondary minima in the ‘reefs’ at low masses has small values of $\tan \beta$. We also see that the preferred values of $\sigma ^\mathrm{SI}_p$ for $\mu < 0$ are $\sim 10^{-44}$ to $10^{-45}$ cm$^2$ at large $m_{\tilde{\chi }^0_{1}}$, whereas $\sigma ^\mathrm{SI}_p$ is $\lesssim 10^{-48}$ cm$^2$ in the ‘reef’ region.

### The NUHM1 with $\mu > 0$

We now turn our attention to the NUHM1, concentrating on the case $\mu > 0$, since our study of the CMSSM indicates that this sign is still preferred by the data, albeit less strongly than in [33].

#### NUHM1 parameter planes

Figure 12 displays our selection of NUHM1 parameter planes, with the same conventions for solid/dashed lines as in Fig. 3. We see in the upper left panel that the likelihood function is relatively flat for $m_{1/2} \gtrsim 2000 \,\, \mathrm {GeV}$, and that there is a low-mass ‘peninsula’ extending down to $(m_0, m_{1/2}) \sim (500, 1200) \,\, \mathrm {GeV}$, which is analogous to the ‘island’ in the CMSSM. The 68 % CL region extends to values of $m_{1/2} > 4000 \,\, \mathrm {GeV}$, which was not the case in the CMSSM. This is because the NUHM1 is able to satisfy the $\Omega _\chi h^2$ constraint for larger values of $m_{1/2}$ than are possible in the CMSSM, thanks to the extra degree of freedom associated with the soft SUSY-breaking contribution to the Higgs masses. This permits values of $\mu$ or $M_A$ that allow $\Omega _\chi h^2$ to fall within the astrophysical range even if $m_{1/2}$ is large. We also note that the NUHM1 can satisfy the electroweak vacuum conditions in regions of the parameter space with $m_0^2 < 0$, though we have not studied this possibility in any detail.

The differences in Fig. 12 between the results of the current analysis (solid lines and filled stars) with our current implementations of the data constraints used in [33] are relatively minor. On the other hand, looking back at the right panel of Fig. 4 where our current NUHM1 results are compared with those shown previously in [33], cf, the dashed lines and open star, we see that both the 68 % and the 95 % CL regions now extend to much larger $m_{1/2}$. This is largely the result of sampling an extended range in $m_H^2$, as well as using FeynHiggs 2.10.0 to calculate $M_h$. As in the CMSSM case shown in the left panel of Fig. 4, the extension of the 95 % CL region to lower $m_{1/2}$ at large $m_0$ is due to the new implementation of the dark matter scattering constraint discussed in Sect. 2.6.

The upper right panel of Fig. 12 displays the $(m_0, \tan \beta )$ plane in the NUHM1. We see a general trend for the preferred range of $\tan \beta$ to increase with the value of $m_0 \gtrsim 1000 \,\, \mathrm {GeV}$. Values of $\tan \beta$ as low as $\sim 5$ are allowed in the ‘peninsula’ region. In the $(\tan \beta , m_{1/2})$ plane shown in the lower left panel of Fig. 12, we see that values of $\tan \beta \sim 5$ to 30 are preferred when $m_{1/2} \lesssim 2000 \,\, \mathrm {GeV}$, whereas larger values of $m_{1/2}$ are associated with $\tan \beta \gtrsim 15$. Finally, we see in the lower right panel of Fig. 12 that values of $M_A\gtrsim 500 \,\, \mathrm {GeV}$ are generally preferred, with most of the favoured region appearing in a lobe with $M_A\gtrsim 2000 \,\, \mathrm {GeV}$.

### Characteristics of the best-fit points in the NUHM1

The best-fit point in the ‘continental’ region has nearly degenerate $\tilde{\chi }^0_{1}$, $\tilde{\chi }^0_{2}$ and $\tilde{\chi }^\pm _1$, since $\mu \ll m_{1/2}$ and the LSP is nearly a pure higgsino, and the $\tilde{\tau }_1$ is $\sim 20 \,\, \mathrm {GeV}$ heavier in this case. Thus $\tilde{\chi }^\pm _1$, $\tilde{\chi }^{0}_{1,2}$ coannihilation is important in fixing $\Omega _\chi h^2$, but $\tilde{\tau }_1$ coannihilation is not negligible. As could be expected from the shape of the 68 % CL region in the lower right panel of Fig. 12, whilst $\tilde{\chi }^\pm _1$, $\tilde{\chi }^0_{2}$ coannihilation is important in most of the ‘continental’ region, different dynamical processes are important in different regions of the NUHM1 parameter space. For example, $\tilde{\tau }_1$ coannihilation and rapid annihilation via direct-channel poles are both important in the lobe where $M_A\sim 1000 \,\, \mathrm {GeV}$ and $\tan \beta \sim 10$, which includes the best-fit point to the previous data set (open star). On the other hand, only rapid annihilation via direct-channel poles is important in the lobe where $M_A\sim 1000 \,\, \mathrm {GeV}$ and $\tan \beta \sim 30$, and only $\tilde{\chi }^\pm _1$, $\tilde{\chi }^0_{2}$ coannihilation is important in the narrow strip where $M_A\sim 1000 \,\, \mathrm {GeV}$ and $\tan \beta \sim 55$. Finally, both $\tilde{\chi }^\pm _1$ coannihilation and rapid annihilation via direct-channel poles are important in the lobe where $M_A\gtrsim 2000 \,\, \mathrm {GeV}$ and $\tan \beta \lesssim 60$.

**Fig. 14:**
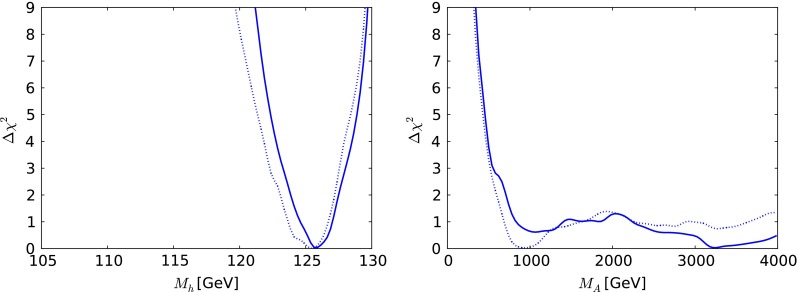
The one-dimensional $\chi ^2$ likelihood functions in the NUHM1 for $\mu > 0$ for $M_h$ (*left*) and $M_A$ (*right*). In each panel, the *solid line* is derived from a global analysis of the present data, and the *dotted line* is derived from a reanalysis of the data used in [33], using the implementations of the $M_h$ and $\sigma ^\mathrm{SI}_p$ constraints discussed in Sect. 2

**Fig. 15:**
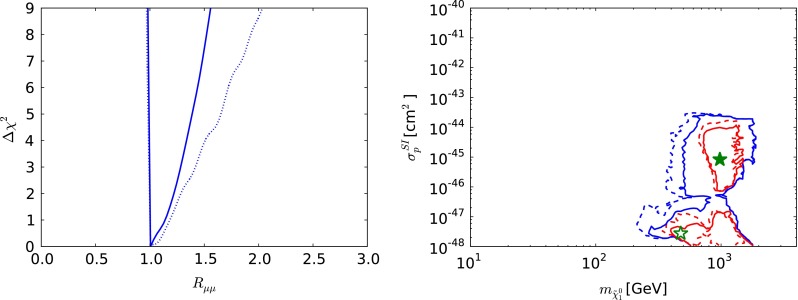
The one-dimensional $\chi ^2$ likelihood function in the NUHM1 for $\mu > 0$ for $\mathrm{BR}(B_{s, d} \rightarrow \mu ^+\mu ^-)$ (*left*) and the $(m_{\tilde{\chi }^0_{1}} , \sigma ^\mathrm{SI}_p)$ plane (*right*). In both panels, the *solid lines* are derived from a global analysis of the present data, and the *dotted lines* are derived from a reanalysis of the data used in [33], using the implementations of the $M_h$ and $\sigma ^\mathrm{SI}_p$ constraints discussed in Sect. 2. In the *right panel*, the *red lines* denote the $\Delta \chi ^2 = 2.30$ contours, the *blue lines* denote the $\Delta \chi ^2 = 5.99$ contours in each case and the *filled* (*open*) *green star* denotes the corresponding best-fit point

We see in Table 2, comparing the contributions to the global $\chi ^2$ functions for the high-mass points in the NUHM1 and the CMSSM with $\mu > 0$, that the NUHM1 point has a noticeably smaller $\chi ^2$ contribution from $M_W$. Comparing the low-mass points in the NUHM1 and the CMSSM with $\mu > 0$, we see that the NUHM1 point has smaller $\chi ^2$ contributions from $\mathrm{BR}(b \rightarrow s \gamma )$ and ATLAS 20/fb jets + ${E\!\!/}_{T}$, in particular. The $M_h$ constraint does not make an important contribution to $\chi ^2$ at either of the NUHM points.

### One-dimensional likelihood functions in the NUHM1

Figure 13 displays the one-dimensional $\chi ^2$ functions for various sparticle masses. The likelihood function for $m_{\tilde{g}}$ (upper left panel) decreases essentially monotonically until $m_{\tilde{g}}\sim 2600 \,\, \mathrm {GeV}$, which is followed by a local maximum at $m_{\tilde{g}}\sim 3500 \,\, \mathrm {GeV}$. The global minimum is at 6800 GeV and hence not visible on this plot. The $\chi ^2$ function for $m_{\tilde{q}_R}$ shown in the upper right panel of Fig. 13 has similar behaviour. On the other hand, the $\chi ^2$ function for $m_{\tilde{t}_1}$, shown in the lower left panel of Fig. 13, manifests an important local minimum at $m_{\tilde{t}_1}\sim 700 \,\, \mathrm {GeV}$ followed by a local maximum at $m_{\tilde{t}_1}\sim 1000 \,\, \mathrm {GeV}$ before exhibiting a second local minimum and local maximum at $m_{\tilde{t}_1}\sim 2000$ and $2700 \,\, \mathrm {GeV}$, respectively. Finally, the $\chi ^2$ function for $m_{\tilde{\tau }_1}$, seen in the lower right panel of Fig. 13, exhibits a low-mass local minimum at $m_{\tilde{\tau }_1}\sim 500 \,\, \mathrm {GeV}$ associated with the above-mentioned ‘peninsula’ followed by a local maximum at $m_{\tilde{\tau }_1}\sim 700 \,\, \mathrm {GeV}$, and then it falls to a shallow minimum at $m_{\tilde{\tau }_1}\sim 1000 \,\, \mathrm {GeV}$, eventually rising slowly at larger masses.

**Fig. 16:**
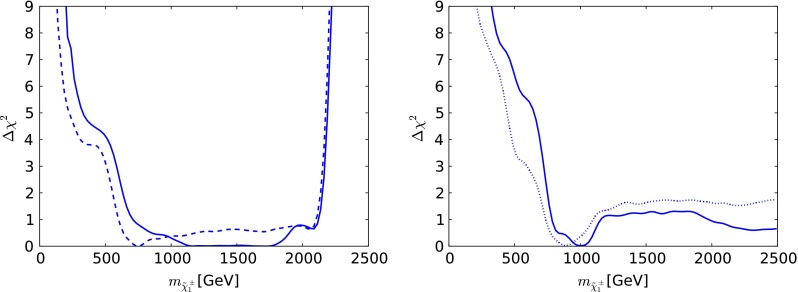
One-dimensional likelihood functions in the CMSSM for $\mu >0$ (*left panel*) and NUHM1 (*right panel*) of the lightest chargino $\tilde{\chi }^\pm _{1}$. In *each panel*, the *solid line* is derived from a global analysis of the present data, and the *dotted line* is obtained from a reanalysis of the data used in [33], using the implementations of the $M_h$ and $\sigma ^\mathrm{SI}_p$ constraints discussed in Sect. 2

Turning now to the one-dimensional $\chi ^2$ functions for the SUSY Higgs bosons shown in Fig. 14, we see in the left panel that the likelihood function for the mass of the lightest supersymmetric Higgs boson $M_h$ is maximised very close to the experimental value, though with tail extending to lower and higher masses reflecting the theoretical uncertainty in the calculation. As for $M_A$, we see in the right panel of Fig. 14 that the likelihood function is rather flat for $M_A\gtrsim 1000 \,\, \mathrm {GeV}$. The 95 % CL lower bounds on $m_{\tilde{g}}, m_{\tilde{q}_R}, m_{\tilde{t}_1}, m_{\tilde{\tau }_1}$ and $M_A$ inferred from the one-dimensional $\chi ^2$ functions in Figs. 13 and 14 are tabulated in Table 3. As in the CMSSM cases studied, the ${\tilde{t}_1}$ may be significantly lighter than the other strongly interacting sparticles.

We see in Fig. 15 that the one-dimensional $\chi ^2$ function for $\mathrm{BR}(B_s \rightarrow \mu ^+\mu ^-)$ is minimised close to the SM value. The NUHM1 offers very little scope for values of $\mathrm{BR}(B_s \rightarrow \mu ^+\mu ^-)$ below this, but values larger than in the SM are not so strongly disfavoured. The right plot of Fig. 15 shows the NUHM1 results in the $(m_{\tilde{\chi }^0_{1}}, \sigma ^\mathrm{SI}_p)$ plane. Similar ranges of $m_{\tilde{\chi }^0_{1}}$ and $\sigma ^\mathrm{SI}_p$ are favoured as for the CMSSM with $\mu > 0$.

Finally we show some in Fig. 16 a comparison of the one-dimensional likelihoods for the mass of the lightest chargino $\tilde{\chi }^\pm _{1}$ for the CMSSM with $\mu >0$ and the NUHM1. (The corresponding likelihood function for the $\tilde{\chi }^0_{2}$ would be similar.) In the CMSSM the $\tilde{\chi }^\pm _{1}$ likelihood function is quite flat between around 800 GeV and 2100 GeV, before rising steeply after that. The NUHM1 likelihood is similar to the CMSSM one below 1000 GeV, but maintains its flatness out to 2500 GeV. This reflects the flatness of the NUHM1 likelihood at out to large values of $m_{1/2}$.

## Summary and prospects

We have presented in this paper analyses of the CMSSM with both signs of $\mu$ and the NUHM1 with $\mu >0$ that take into account all the relevant constraints from the first run of the LHC with $\sim 5$/fb of luminosity at 7 TeV and $\sim 20$/fb of luminosity at 8 TeV, as well as flavour and precision electroweak observables and the first results from the LUX search for spin-independent dark matter scattering [9]. We have sampled the model parameter spaces using the MultiNest technique, made SUSY model calculations of $M_h$ using version 2.10.0 of the FeynHiggs code, and we have taken account of uncertainties in these calculations and in the estimation of hadronic matrix elements for dark matter scattering.

It is a general feature of our analysis that we find larger values of $m_0$ and $m_{1/2}$ to be allowed than were found in our previous analyses, largely because of our updated interpretation of the experimental $M_h$ constraint using FeynHiggs 2.10.0 and the newer version of MicrOMEGAs that we use. The parameters of the best fits we find in the CMSSM and NUMH1 are displayed in Table 1: we note that they also have larger values of $m_0$ and $m_{1/2}$ than were favoured previously. Also shown for comparison are the model parameters for local minima of the global $\chi ^2$ functions at low masses, which are disfavoured by the ATLAS 20/fb jets + ${E\!\!/}_{T}$ constraint, in particular. We note that all the favoured CMSSM and NUHM1 model points can accommodate the measured value of $M_h$. None of the SUSY models studied has a global $\chi ^2$ value that is much lower than the SM. This is because none of the SUSY models discussed reduces significantly the contributions to the global $\chi ^2$ functions from the observables that make the largest contributions to the global $\chi ^2$ functions in the SM fit, namely $(g-2)_\mu$, $A_\mathrm{fb}({b})$, $A_\ell (\mathrm{SLD})$, $\sigma _\mathrm{had}^0$, $\epsilon _K$ and $\mathrm{BR}(B_u \rightarrow \tau \nu _\tau )$, as seen in Table 2.

The 95 % CL lower limits on sparticle masses found in our CMSSM and NUHM1 analysis are displayed in Table 3. We see that gluino masses above $\sim 1300 \,\, \mathrm {GeV}$ are preferred in the models analyzed. The right-handed squark mass is restricted to even higher values, because of the preferred values of $m_0$, whereas the lighter stop squark may be significantly lighter. The lighter stau slepton may also be relatively light in the CMSSM and NUHM1 with $\mu > 0$. On the other hand, the heavier Higgs bosons $A, H$ and $H^\pm$ are all expected to have masses above $500 \,\, \mathrm {GeV}$ in these models.

Estimates of the discovery reach of the LHC at 14 TeV have been provided in [126]. With 300/fb of luminosity, the 5-$\sigma$ discovery reach for squarks and gluinos should extend to $(m_{\tilde{q}_R}, m_{\tilde{g}}) \sim (3500, 2000)$ GeV in the CMSSM with $\mu > 0$, would include the low-mass ‘reef’ in the CMSSM with $\mu < 0$, and would reach the first local minimum of the $\chi ^2$ function in the NUHM1 with $\mu > 0$, at $(m_{\tilde{q}_R}, m_{\tilde{g}}) \sim (2500, 3000)$ GeV. The discovery range with 3000/fb of luminosity would extend a few hundred GeV further, and it would be very similar to the 95 % CL exclusion reach with 300/fb. The reach for 95 % CL exclusion with 3000/fb would extend several hundred GeV further still, e.g., to $(m_{\tilde{q}_R}, m_{\tilde{g}}) \sim (4000, 2700)$ GeV in the CMSSM with $\mu > 0$.

We conclude that large parts of the preferred parameter regions of the CMSSM and NUHM1 are accessible in future runs of the LHC, although the strongly interacting sparticle masses might be so high as to escape the searches at the LHC. That said, we re-emphasise that all the likelihood estimates made in this paper and the estimates of the LHC physics reach are specific to the models studied, and are quite model dependent. The approach we have followed here for constructing the global likelihood function can easily be extended to other models, a subject to which we will turn in future work.

## Footnotes

1 Special relations between sparticle masses are required to achieve the correct relic density, and relaxing this requirement to use the relic density only as an upper limit would not greatly affect our results.

2 In this analysis we use the estimate $\Delta \alpha _\mathrm{had} (M_Z) = 0.002756 \pm 0.0010$ [73].

3 We use the same convention for the sign of $A_0$ as in [33], which is opposite to the convention used in SoftSUSY.

4 This version was an extension of the publicly available FeynHiggs 2.8.6, which differed in the conversion of the trilinear coupling $A_b$ from the $\overline{\mathrm {DR}}$ scheme to the on-shell (OS) scheme. This issue was treated in an improved way in FeynHiggs 2.9.5. The implementation of FeynHiggs used here slightly differs from the public FeynHiggs 2.10.0, with a small difference of $\lesssim 0.5 \,\, \mathrm {GeV}$ in the $M_h$ calculation.

5 The results from the ATLAS [100], CDF [101] and DØ [102] Collaborations are not considered in our study, as they have significantly less precision than the results of CMS and LHCb.

6 The numerical value in (6) is obtained taking into account the latest SM inputs from Ref. [103–105].

7 We estimate this as in [33], i.e., by using MasterCode to calculate the $\chi ^2$ for the CMSSM point with $(m_0, m_{1/2}) = 15$ TeV, $\tan \beta = 10$ and $A_0 = 100 \,\, \mathrm {GeV}$, discarding the contributions from $M_h$ and $\Omega _\chi h^2$ and evaluating the $\sigma ^\mathrm{SI}_p$ contribution as 0.14, which yields $\chi ^2 = 36.5$.

8 We discuss later the best-fit points for the CMSSM with $\mu < 0$ and for the NUHM1 with $\mu > 0$.

9 The fact that the light CMSSM Higgs boson should be SM-like was already a *pre*-LHC prediction of the model [122, 123].

10 However, adding many channels of Higgs production and decay properties whose measurements agree with the predictions for a SM Higgs boson would yield a better $\chi ^2/\mathrm{dof}$.

11 Here and in subsequent panels, we restrict attention to $\tan \beta \le 40$. The parameters required to solve the electroweak vacuum conditions for larger values of $\tan \beta$ are relatively unstable, often with values of $m_0$ and $A_0$ outside the ranges studied here, so the limits on the ranges of these parameters that we sample imply that our sampling is incomplete for $\tan \beta > 40$ and $\mu < 0$.

12 As in the $\mu > 0$ case, we have not studied in detail the sensitivity to $\tan \beta$ of the lower bound on $m_{1/2}$ due to ATLAS 20/fb jets + ${E\!\!/}_{T}$ searches with leptons and/or $b$ jets, which are not important near the ‘reef’ or the best-fit point for $\mu < 0$.

13 However, our incomplete sampling at larger $\tan \beta$ shows that $M_A\sim 1000 \,\, \mathrm {GeV}$ is allowed for $\tan \beta \sim 50$.
